# Establishment and validation of highly accurate formalin-fixed paraffin-embedded quantitative proteomics by heat-compatible pressure cycling technology using phase-transfer surfactant and SWATH-MS

**DOI:** 10.1038/s41598-020-68245-2

**Published:** 2020-07-09

**Authors:** Yasuo Uchida, Hayate Sasaki, Tetsuya Terasaki

**Affiliations:** Division of Membrane Transport and Drug Targeting, Graduate School of Pharmaceutical Sciences, Tohoku University, Sendai, Japan

**Keywords:** Proteomic analysis, Diagnostic markers

## Abstract

The purpose of this study was to establish a quantitative proteomic method able to accurately quantify pathological changes in the protein expression levels of not only non-membrane proteins, but also membrane proteins, using formalin-fixed paraffin-embedded (FFPE) samples. Protein extraction from FFPE sections of mouse liver was increased 3.33-fold by pressure cycling technology (PCT) and reached the same level as protein extraction from frozen sections. After PCT-assisted processing of FFPE liver samples followed by SWATH-MS-based comprehensive quantification, the peak areas of 88.4% of peptides agreed with those from matched fresh samples within a 1.5-fold range. For membrane proteins, this percentage was remarkably increased from 49.1 to 93.8% by PCT. Compared to the conventional method using urea buffer, the present method using phase-transfer surfactant (PTS) buffer at 95 °C showed better agreement of peptide peak areas between FFPE and fresh samples. When our method using PCT and PTS buffer at 95 °C was applied to a bile duct ligation (BDL) disease model, the BDL/control expression ratios for 80.0% of peptides agreed within a 1.2-fold range between FFPE and fresh samples. This heat-compatible FFPE-PCT-SWATH proteomics technology using PTS is suitable for quantitative studies of pathological molecular mechanisms and biomarker discovery utilizing widely available FFPE samples.

## Introduction

One of the key issues in proteomics-based studies to explore tissue biomarkers and pathological molecular mechanisms is that the number of specimens is limited^1^, because fresh or frozen tissues are usually used for proteomics experiments. On the other hand, formalin-fixed paraffin-embedded (FFPE) specimens can be preserved at room temperature for long period, unlike fresh or frozen tissue specimens. Further, many such specimens are stored in medical institutes, together with the corresponding clinical history, and they are routinely used for pathological diagnosis. Thus, in order to clarify the diversity of pathological molecular mechanisms among patients, it is highly desirable to use FFPE clinical specimens in proteomics-based studies. However, the presence of crosslinking among proteins induced by formalin fixation hampers the establishment of FFPE-based proteomics^2^.

In proteomics studies using FFPE specimens, several thousand proteins have so far been identified with over 90% overlap between them and matched frozen samples in terms of coverage^3^, but it remains unclear how well the protein expression levels determined by using FFPE specimens agree quantitatively with those obtained by using matched fresh tissues. Good agreement has been established for some proteins, but this is limited to non-membrane proteins such as cytosolic proteins^4^. Thus, we still require a proteomics technique to accurately and comprehensively quantify disease-associated changes of protein expression levels by using FFPE specimens.

Pressure cycling technology (PCT) is a sample-processing technique that employs a rapidly alternating hydrostatic pressure change between ambient (14.7 psi) and high pressure (up to 45,000 psi) within a 3 s rise time and millisecond pressure-drop time. It is expected to promote de-crosslinking, extraction and trypsin digestion of proteins from FFPE sections; indeed, it has been reported that PCT improves the number of proteins identified in shotgun proteomics from FFPE samples^5^. In addition to PCT-assisted sample processing, a quantitatively accurate LC–MS/MS analysis is also needed to identify disease-associated changes in protein expression levels. Sequential window acquisition of all theoretical fragment ion spectra–mass spectrometry (SWATH-MS) is the latest quantitative comprehensive proteomics method, and is highly accurate and reproducible compared to conventional shotgun proteomics^6^.

Zhu et al. have performed PCT-assisted sample processing followed by SWATH-MS analysis of FFPE specimens, and found that the overall technical CV value is similar to that of matched frozen tissue, and there is a fairly good correlation between the protein abundances found in FFPE and frozen specimens (Pearson R^2^ = 0.83)^7^. However, they did not clarify the extent to which PCT-assisted sample processing and SWATH-MS analysis are superior to conventional FFPE proteomics, or how well their FFPE proteomics method quantitatively reflects the disease-associated changes in protein expression levels observed in fresh tissues. Furthermore, to completely de-crosslink and extract proteins from FFPE specimens, a combination of PCT and heat (around 95 °C) in the sample processing is thought to be necessary. However, Zhu et al. used urea as a lysis buffer, so they were not able to heat the samples^7^, because urea reacts and binds with proteins in the presence of heat^8,9^. Phase-transfer surfactant (PTS), such as sodium deoxycholate (SDC) or sodium lauroylsarcosinate (SLS), can lyse proteins much more effectively than urea, and is resistant to heat^10^. Therefore, we hypothesized that the use of PTS buffer would overcome the above issue and facilitate the complete de-crosslinking and extraction of proteins from FFPE specimens.

Therefore, the aim of the present study was to quantitatively establish the superiority of PCT-assisted sample processing with PTS buffer/heat followed by SWATH-MS analysis over conventional FFPE proteomics methodology. We also confirmed that the present FFPE-PCT-SWATH proteomics protocol can accurately reflect the disease-associated changes of protein expression levels observed in fresh tissues.

## Materials and methods

### Normal and bile duct ligation (BDL) mice

Details of the mice used and the preparation of bile duct ligation (BDL) mice are given in the Supplementary Methods. All experimental protocols were approved by the Institutional Animal Care and Use Committee in Tohoku University, and were performed in accordance with the guidelines of Tohoku University.

### Preparation of FFPE tissue sections of mouse liver

Under anesthesia induced with isoflurane, mice were transcardially perfused first with phosphate-buffered saline (PBS) (137 mM NaCl, 2.7 mM KCl, 10 mM Na_2_HPO_4_, 1.8 mM KH_2_PO_4_, pH 7.4) and then with 4% paraformaldehyde (PFA) (Merck, Darmstadt, Germany) solution after blood clearance. The liver was immediately isolated, immersed in 20–25 mL of 4% PFA solution and fixed for 24 h at 4 °C. To embed a fixed sample in paraffin, tissues were equilibrated in turn in 70% (v/v) ethanol (1 h), then 80% ethanol (1 h), 90% ethanol (1 h), 99% ethanol (2 × 1 h), absolute ethanol (1 h and overnight), xylene (3 × 1 h), and paraffin (3 × 1.5 h) (Wako Pure Chemical Industries, Osaka, Japan). Embedded samples were sliced in a microtome to obtain 5 μm sections, which were mounted on standard slides.

### Preparation of FFPE tissue suspension of mouse liver

Slide-mounted FFPE tissue sections were incubated in xylene (3 × 5 min), then absolute ethanol (2 × 1 min), 95% (v/v) ethanol (2 × 1 min), 70% ethanol (2 × 1 min), and Milli-Q water (1 min). After air-drying, 60 sections of deparaffinized tissue were harvested with a microtome blade into a 15 mL low-protein-adsorption centrifuge tube, and suspended by sonication in 7.2 mL of phase-transfer surfactant (PTS) (10 mM sodium deoxycholate (SDC), 10 mM sodium lauroyl sarcosinate (SLS) (Sigma-Aldrich St. Louis, MO), 0.1 M Tris–HCl, pH 9.0) buffer. The suspension was stored at 4 °C until use.

### Preparation of fresh tissue lysate samples of mouse liver

Fresh tissue lysate samples were prepared as previously reported^11,12^. All procedures were carried out at 4 °C. Perfusion with PBS was performed in the same way as described for the FFPE samples, but without 4% PFA. The isolated fresh liver was immediately cut into pieces and suspended in hypotonic buffer (10 mM Tris–HCl, 10 mM NaCl, 1.5 mM MgCl_2_, pH 7.4) with phenylmethylsulfonyl fluoride (PMSF), and protease inhibitor cocktail (Sigma-Aldrich, St. Louis, MO). Samples were homogenized with a Potter–Elvehjem homogenizer followed by nitrogen cavitation (450 psi for 15 min at 4 °C). Protein concentrations of the lysates were measured by means of the Lowry assay, and the lysates were stored at − 80 °C until use.

### Evaluation of PCT-assisted protein extraction from FFPE sections

The effect of PCT treatment on protein extraction from FFPE sections was evaluated as described in Supplementary Methods.

### Protein extraction from FFPE and fresh liver with or without PCT treatment to prepare protein samples for SWATH and shotgun analysis using LC–MS/MS

FFPE tissue suspension and fresh whole-tissue lysate of mouse liver, prepared as described above, were placed in PCT Micro Tubes with PCT Micro Caps (100 μL size) to make about 120 μg protein in 120 μL PTS buffer per tube (based on the protein amounts extracted by PCT-assisted lysis). All samples were incubated at 95 °C for 60 min in a block incubator (Eppendorf, Hamburg, Germany; with mixing at 1,000 rpm). Thereafter, both FFPE and fresh samples were processed with or without PCT treatment. For PCT treatment, samples were incubated in a Barocycler (NEP 2,320 Enhanced; Pressure BioSciences, South Easton, MA) in two steps: firstly, 60 cycles of 95 s at 45,000 psi and 5 s at atmospheric pressure at 95 °C, and secondly, 50 cycles of 20 s at 45,000 psi and 15 s at atmospheric pressure at 95 °C. Samples without PCT treatment were simply incubated in a block incubator (Eppendorf, Hamburg, Germany; without mixing) at 95 °C for the same time as used for the PCT treatment. In the case of FFPE samples incubated without PCT, the amount of extracted protein was not sufficient to perform LC–MS/MS measurement, so in these cases, we prepared twice as many 120 μL FFPE suspension tubes and incubated them as described above.

### Protein digestion of FFPE and fresh liver samples with or without PCT treatment

Protein digestion was carried out as soon as possible after protein extraction. After centrifuging the above samples at 15,000 rpm and room temperature for 3 min, 40 μL aliquots of the supernatants were transferred to new PCT Micro Tubes with PCT Micro Caps (150 μL size) (Pressure BioSciences, South Easton, MA). Samples were reduced in 10 mM (±)-dithiothreitol (DTT) (Wako Pure Chemical Industries, Osaka, Japan) for 30 min at 25 °C, followed by alkylation with 40 mM iodoacetamide (IAA) (Wako Pure Chemical Industries, Osaka, Japan) in the dark at 25 °C. Samples were diluted by adjusting the buffer volume to 139 μL with 50 mM ammonium bicarbonate before addition of Protease Max surfactant (Promega, Madison, WI) and Lys-C (Wako Pure Chemical Industries, Osaka, Japan) at 0.04% final concentration and an enzyme/substrate ratio of 1:20, respectively. PCT-assisted Lys-C digestion was performed in the Barocycler at 37 °C using 60 cycles of 50 s at 45,000 psi, and 10 s at atmospheric pressure, while Lys-C digestion without PCT was performed for 3 h at 37 °C. Subsequently, trypsin (Promega, Madison, WI) was added at an enzyme/substrate ratio of 1:20 with Protease Max surfactant. PCT-assisted trypsin digestion was performed in the Barocycler at 37 °C using 90 cycles of 50 s at 45,000 psi (20,000 psi for fresh samples), and 10 s at atmospheric pressure, while trypsin digestion without PCT was performed for 16 h at 37 °C. After enzyme digestion, SDC and SLS (PTS) were removed by liquid–liquid extraction using ethyl acetate, as previously reported^13^. The aqueous phase was dried under vacuum and stored at − 80 °C until desalting. Peptide samples dissolved in 0.1% trifluoroacetic acid/water were desalted with self-packed SDB-XD tips (3 M, Maplewood, MN), and the eluted peptide solution was dried under vacuum again. Peptides samples were dissolved in 0.1% formic acid/2% acetonitrile/98% water, and their concentration was adjusted to 0.5 μg/μL based on the BCA assay. As described for FFPE, in the case of samples to be incubated without PCT, 2 tubes of the same sample were combined to make one sample when desalting.

### LC–MS/MS measurement in the SWATH and shotgun modes

LC–MS/MS measurement in the SWATH or shotgun mode was performed with 1 μg peptide injection as previously reported^12,14^. The SWATH and shotgun chromatograms were uploaded to the PeptideAtlas webpage with identifiers PASS01457 and PASS01463, respectively. The spectral library for SWATH analysis was generated as described in the Supplementary Methods and includes 3,140 proteins.

### Analysis of SWATH and shotgun data

In data extraction from SWATH chromatograms, the peaks were identified and quantified using PeakView 2.2 software (SCIEX, Framingham, MA, USA) with the spectral library (3,140 proteins) generated as described in the Supplementary Methods. The conditions for data extraction in PeakView2.2 software are described in “Data extraction (left side)” of Supplementary Fig. 2. According to the criteria described in “Data selection procedure (left side)” in Supplementary Fig. 2, reliable data were selected from all the transitions extracted from SWATH chromatograms and each peak area was calculated at the peptide level. On the other hand, data extraction from shotgun chromatograms was performed using Skyline software (Version 4.1, freely available, open-source Windows client application) with the UniProt Mouse proteome database (release2018_03, entries), which was uploaded to the PeptideAtlas webpage with the identifier PASS01463. The data extraction conditions used in Skyline software are described in “Data extraction (right side)” of Supplementary Fig. 2. According to the criteria described in “Data selection procedure (right side)” in Supplementary Fig. 2, reliable data were selected from all the data extracted from shotgun chromatograms.

After the basic data selection above, reliable peptides were selected according to the “Peptide selection criteria” in Supplementary Fig. 2 both for SWATH and shotgun data, and then used for figure generation, such as the comparison of FFPE and fresh samples. The comparison of peak areas between FFPE and fresh samples in PCT(+)-SWATH was conducted not only at the peptide level, but also at the protein level, calculated as average values of the relevant peptide peak areas.

### Calculation of inaccuracy and CV values

Inaccuracy of peak area for each peptide was calculated as the difference between the mean peak area from FFPE and the mean peak area from the fresh sample, divided by the mean peak area from the fresh sample. The CV value of the peak area for each peptide among four independent sample preparations was calculated for the FFPE samples as shown below. The experimental design including randomization is shown in Supplementary Table 1.

$$\mathrm{I}\mathrm{n}\mathrm{a}\mathrm{c}\mathrm{c}\mathrm{u}\mathrm{r}\mathrm{a}\mathrm{c}\mathrm{y} \, \mathrm{o}\mathrm{f} \mathrm{p}\mathrm{e}\mathrm{a}\mathrm{k} \,  \mathrm{a}\mathrm{r}\mathrm{e}\mathrm{a} \, \mathrm{f}\mathrm{o}\mathrm{r} \,  \mathrm{e}\mathrm{a}\mathrm{c}\mathrm{h} \,  \mathrm{p}\mathrm{e}\mathrm{p}\mathrm{t}\mathrm{i}\mathrm{d}\mathrm{e}\left(\mathrm{\%}\right)=\frac{|\mathrm{T}\mathrm{h}\mathrm{e}  \, \mathrm{m}\mathrm{e}\mathrm{a}\mathrm{n} \mathrm{o}\mathrm{f} \mathrm{p}\mathrm{e}\mathrm{a}\mathrm{k}  \,  \mathrm{a}\mathrm{r}\mathrm{e}\mathrm{a} \,  \mathrm{f}\mathrm{r}\mathrm{o}\mathrm{m}  \,  \mathrm{f}\mathrm{r}\mathrm{e}\mathrm{s}\mathrm{h}  \,  \mathrm{s}\mathrm{a}\mathrm{m}\mathrm{p}\mathrm{l}\mathrm{e}\mathrm{s} -\mathrm{T}\mathrm{h}\mathrm{e}  \,  \mathrm{m}\mathrm{e}\mathrm{a}\mathrm{n} \,  \mathrm{o}\mathrm{f}  \, \mathrm{p}\mathrm{e}\mathrm{a}\mathrm{k} \,  \mathrm{a}\mathrm{r}\mathrm{e}\mathrm{a}  \, \mathrm{f}\mathrm{r}\mathrm{o}\mathrm{m} \mathrm{F}\mathrm{F}\mathrm{P}\mathrm{E} \,  \mathrm{s}\mathrm{a}\mathrm{m}\mathrm{p}\mathrm{l}\mathrm{e}\mathrm{s}| }{\mathrm{T}\mathrm{h}\mathrm{e}  \,  \mathrm{m}\mathrm{e}\mathrm{a}\mathrm{n} \mathrm{o}\mathrm{f}  \,  \mathrm{p}\mathrm{e}\mathrm{a}\mathrm{k}  \,  \mathrm{a}\mathrm{r}\mathrm{e}\mathrm{a} \mathrm{f}\mathrm{r}\mathrm{o}\mathrm{m} \mathrm{f}\mathrm{r}\mathrm{e}\mathrm{s}\mathrm{h}  \,  \mathrm{s}\mathrm{a}\mathrm{m}\mathrm{p}\mathrm{l}\mathrm{e}\mathrm{s} }\times 100$$

$$\mathrm{C}\mathrm{V}  \,  \mathrm{v}\mathrm{a}\mathrm{l}\mathrm{u}\mathrm{e}   \,  \mathrm{o}\mathrm{f}  \,  \mathrm{p}\mathrm{e}\mathrm{a}\mathrm{k}  \,  \mathrm{a}\mathrm{r}\mathrm{e}\mathrm{a}  \,  \mathrm{f}\mathrm{o}\mathrm{r}  \,  \mathrm{e}\mathrm{a}\mathrm{c}\mathrm{h} \,   \mathrm{p}\mathrm{e}\mathrm{p}\mathrm{t}\mathrm{i}\mathrm{d}\mathrm{e}  \,  \mathrm{i}\mathrm{n}  \,  \mathrm{F}\mathrm{F}\mathrm{P}\mathrm{E} \,   \mathrm{s}\mathrm{a}\mathrm{m}\mathrm{p}\mathrm{l}\mathrm{e}\mathrm{s} \left(\mathrm{\%}\right)=\frac{\mathrm{T}\mathrm{h}\mathrm{e}  \,  \mathrm{S}\mathrm{D} \mathrm{v}\mathrm{a}\mathrm{l}\mathrm{u}\mathrm{e}  \,  \mathrm{o}\mathrm{f} \mathrm{p}\mathrm{e}\mathrm{a}\mathrm{k} \mathrm{a}\mathrm{r}\mathrm{e}\mathrm{a} \mathrm{a}\mathrm{m}\mathrm{o}\mathrm{n}\mathrm{g}  \,  \mathrm{f}\mathrm{o}\mathrm{u}\mathrm{r}  \,  \mathrm{i}\mathrm{n}\mathrm{d}\mathrm{e}\mathrm{p}\mathrm{e}\mathrm{n}\mathrm{d}\mathrm{e}\mathrm{n}\mathrm{t}  \,  \mathrm{p}\mathrm{r}\mathrm{e}\mathrm{p}\mathrm{a}\mathrm{r}\mathrm{a}\mathrm{t}\mathrm{i}\mathrm{o}\mathrm{n}\mathrm{s}  \,  \mathrm{o}\mathrm{f}  \,  \mathrm{F}\mathrm{F}\mathrm{P}\mathrm{E}  \,  \mathrm{s}\mathrm{a}\mathrm{m}\mathrm{p}\mathrm{l}\mathrm{e}\mathrm{s}}{\mathrm{T}\mathrm{h}\mathrm{e} \,  \mathrm{m}\mathrm{e}\mathrm{a}\mathrm{n}  \, \mathrm{o}\mathrm{f} \mathrm{p}\mathrm{e}\mathrm{a}\mathrm{k}  \,  \mathrm{a}\mathrm{r}\mathrm{e}\mathrm{a}  \,  \mathrm{o}\mathrm{f} \,   \mathrm{f}\mathrm{o}\mathrm{u}\mathrm{r} \,  \mathrm{i}\mathrm{n}\mathrm{d}\mathrm{e}\mathrm{p}\mathrm{e}\mathrm{n}\mathrm{d}\mathrm{e}\mathrm{n}\mathrm{t}  \,  \mathrm{p}\mathrm{r}\mathrm{e}\mathrm{p}\mathrm{a}\mathrm{r}\mathrm{a}\mathrm{t}\mathrm{i}\mathrm{o}\mathrm{n}\mathrm{s}  \,  \mathrm{o}\mathrm{f} \mathrm{F}\mathrm{F}\mathrm{P}\mathrm{E}  \,  \mathrm{s}\mathrm{a}\mathrm{m}\mathrm{p}\mathrm{l}\mathrm{e}\mathrm{s}}\times 100$$

Finally, the inaccuracy and CV values under each experimental condition (PCT(+)-SWATH, PCT(−)-SWATH, PCT(+)-Shotgun, etc.) were calculated as the mean ± SEM of all detected peptides.

### Calculation of BDL/control ratio, and its inaccuracy and CV values

To evaluate the agreement between the BDL-induced changes in protein expression level (BDL/control ratio) determined by using FFPE samples and fresh samples, data analysis was performed according to the procedure illustrated in Supplementary Fig. 3. Because the quantitative accuracy is considered high for peptides whose peptide peak areas are similar for FFPE and fresh samples, we thought that we would be able to accurately determine the BDL/control ratio by using these peptides to calculate it. Therefore, the peptides whose peptide peak areas are identical within a 1.2-fold range between FFPE and fresh samples (1.2-fold criterion) were extracted. However, if only the peptides that satisfy the 1.2-fold criterion both with and without PCT treatment (PCT(+) and PCT(−), respectively) are used, the effect of PCT cannot be properly evaluated. Therefore, if peptides satisfying the 1.2-fold criterion under the PCT(+) condition were detected under the PCT(−) condition as well, they were also extracted in the PCT(−) data even if they did not satisfy the 1.2-fold criteria under the PCT(−) condition. To statistically properly evaluate the data without any bias between PCT( +) and PCT(−) conditions, if peptides satisfying the 1.2-fold criteria under the PCT(−) condition were detected under the PCT(+) condition as well, they were also extracted in the PCT(+) data even if they did not satisfy the 1.2-fold criteria under the PCT(+) condition. Then, the peptide peak area in BDL mice was divided by that in control mice to calculate the BDL/control ratio for each peptide, and the BDL/control ratio at the peptide level was used for figure generation, such as the comparison of FFPE and fresh samples. The comparison of BDL/control ratios between FFPE and fresh samples under the PCT(+)-SWATH condition was conducted not only at the peptide level, but also at the protein level. The peak areas at the protein level were calculated as average values of the relevant peptide peak areas just before calculating the BDL/control ratios (Supplementary Fig. 3), and then used to calculate the BDL/control ratios at the protein level.

Inaccuracy and CV values (four independent sample preparations) of the BDL/control ratio for each peptide were calculated as described below. The experimental design including randomization is shown in Supplementary Table 1.

$$\mathrm{I}\mathrm{n}\mathrm{a}\mathrm{c}\mathrm{c}\mathrm{u}\mathrm{r}\mathrm{a}\mathrm{c}\mathrm{y}  \,  \mathrm{o}\mathrm{f} \mathrm{B}\mathrm{D}\mathrm{L}/\mathrm{c}\mathrm{o}\mathrm{n}\mathrm{t}\mathrm{r}\mathrm{o}\mathrm{l}  \,  \mathrm{r}\mathrm{a}\mathrm{t}\mathrm{i}\mathrm{o}  \,  \mathrm{f}\mathrm{o}\mathrm{r}  \,  \mathrm{e}\mathrm{a}\mathrm{c}\mathrm{h} \mathrm{p}\mathrm{e}\mathrm{p}\mathrm{t}\mathrm{i}\mathrm{d}\mathrm{e}\left(\mathrm{\%}\right)=\frac{|\mathrm{T}\mathrm{h}\mathrm{e} \,  \mathrm{m}\mathrm{e}\mathrm{a}\mathrm{n} \mathrm{o}\mathrm{f} \,  \mathrm{B}\mathrm{D}\mathrm{L}/\mathrm{c}\mathrm{o}\mathrm{n}\mathrm{t}\mathrm{r}\mathrm{o}\mathrm{l}  \, \mathrm{r}\mathrm{a}\mathrm{t}\mathrm{i}\mathrm{o}  \,  \, \mathrm{f}\mathrm{r}\mathrm{o}\mathrm{m} \mathrm{f}\mathrm{r}\mathrm{e}\mathrm{s}\mathrm{h}  \, \mathrm{s}\mathrm{a}\mathrm{m}\mathrm{p}\mathrm{l}\mathrm{e}\mathrm{s} -\mathrm{T}\mathrm{h}\mathrm{e} \mathrm{m}\mathrm{e}\mathrm{a}\mathrm{n} \mathrm{o}\mathrm{f}  \, \mathrm{B}\mathrm{D}\mathrm{L}/\mathrm{c}\mathrm{o}\mathrm{n}\mathrm{t}\mathrm{r}\mathrm{o}\mathrm{l}  \, \mathrm{r}\mathrm{a}\mathrm{t}\mathrm{i}\mathrm{o} \mathrm{f}\mathrm{r}\mathrm{o}\mathrm{m} \mathrm{F}\mathrm{F}\mathrm{P}\mathrm{E}  \, \mathrm{s}\mathrm{a}\mathrm{m}\mathrm{p}\mathrm{l}\mathrm{e}\mathrm{s}| }{\mathrm{T}\mathrm{h}\mathrm{e}  \, \mathrm{m}\mathrm{e}\mathrm{a}\mathrm{n} \mathrm{o}\mathrm{f} \,  \mathrm{B}\mathrm{D}\mathrm{L}/\mathrm{c}\mathrm{o}\mathrm{n}\mathrm{t}\mathrm{r}\mathrm{o}\mathrm{l}  \, \mathrm{r}\mathrm{a}\mathrm{t}\mathrm{i}\mathrm{o}  \, \mathrm{f}\mathrm{r}\mathrm{o}\mathrm{m}  \, \mathrm{f}\mathrm{r}\mathrm{e}\mathrm{s}\mathrm{h} \mathrm{s}\mathrm{a}\mathrm{m}\mathrm{p}\mathrm{l}\mathrm{e}\mathrm{s} }\times 100$$

$$\mathrm{C}\mathrm{V}  \, \mathrm{v}\mathrm{a}\mathrm{l}\mathrm{u}\mathrm{e}  \, \mathrm{o}\mathrm{f} \,  \mathrm{B}\mathrm{D}\mathrm{L}/\mathrm{c}\mathrm{o}\mathrm{n}\mathrm{t}\mathrm{r}\mathrm{o}\mathrm{l} \,  \mathrm{r}\mathrm{a}\mathrm{t}\mathrm{i}\mathrm{o}  \, \mathrm{f}\mathrm{o}\mathrm{r}  \, \mathrm{e}\mathrm{a}\mathrm{c}\mathrm{h} \,  \mathrm{p}\mathrm{e}\mathrm{p}\mathrm{t}\mathrm{i}\mathrm{d}\mathrm{e}\left(\mathrm{\%}\right)=\frac{\mathrm{T}\mathrm{h}\mathrm{e} \mathrm{S}\mathrm{D}  \, \mathrm{v}\mathrm{a}\mathrm{l}\mathrm{u}\mathrm{e} \, \mathrm{o}\mathrm{f} \mathrm{B}\mathrm{D}\mathrm{L}/\mathrm{c}\mathrm{o}\mathrm{n}\mathrm{t}\mathrm{r}\mathrm{o}\mathrm{l}  \,  \mathrm{r}\mathrm{a}\mathrm{t}\mathrm{i}\mathrm{o}  \, \mathrm{a}\mathrm{m}\mathrm{o}\mathrm{n}\mathrm{g}  \, \mathrm{f}\mathrm{o}\mathrm{u}\mathrm{r}  \, \mathrm{i}\mathrm{n}\mathrm{d}\mathrm{e}\mathrm{p}\mathrm{e}\mathrm{n}\mathrm{d}\mathrm{e}\mathrm{n}\mathrm{t}  \, \mathrm{p}\mathrm{r}\mathrm{e}\mathrm{p}\mathrm{a}\mathrm{r}\mathrm{a}\mathrm{t}\mathrm{i}\mathrm{o}\mathrm{n}\mathrm{s}  \, \mathrm{o}\mathrm{f}  \, \mathrm{F}\mathrm{F}\mathrm{P}\mathrm{E}  \, \mathrm{s}\mathrm{a}\mathrm{m}\mathrm{p}\mathrm{l}\mathrm{e}\mathrm{s}}{\mathrm{T}\mathrm{h}\mathrm{e}  \, \mathrm{m}\mathrm{e}\mathrm{a}\mathrm{n} \mathrm{o}\mathrm{f}  \, \mathrm{B}\mathrm{D}\mathrm{L}/\mathrm{c}\mathrm{o}\mathrm{n}\mathrm{t}\mathrm{r}\mathrm{o}\mathrm{l}  \, \mathrm{r}\mathrm{a}\mathrm{t}\mathrm{i}\mathrm{o}  \, \mathrm{o}\mathrm{f} \mathrm{f}\mathrm{o}\mathrm{u}\mathrm{r} \,  \mathrm{i}\mathrm{n}\mathrm{d}\mathrm{e}\mathrm{p}\mathrm{e}\mathrm{n}\mathrm{d}\mathrm{e}\mathrm{n}\mathrm{t}  \, \mathrm{p}\mathrm{r}\mathrm{e}\mathrm{p}\mathrm{a}\mathrm{r}\mathrm{a}\mathrm{t}\mathrm{i}\mathrm{o}\mathrm{n}\mathrm{s}  \,  \, \mathrm{o}\mathrm{f} \,  \mathrm{F}\mathrm{F}\mathrm{P}\mathrm{E} \,  \mathrm{s}\mathrm{a}\mathrm{m}\mathrm{p}\mathrm{l}\mathrm{e}\mathrm{s}}\times 100$$

Finally, inaccuracy and CV values under each experimental condition (PCT(+)-SWATH, PCT(-)-SWATH, PCT(+)-Shotgun, etc.) were calculated as the mean ± SEM of all peptides examined. The SD value of the BDL/control ratio among four independent preparations of FFPE samples was calculated according to the law of propagation of error, as previously reported^15^.

### Statistical analysis

All data are presented as the mean ± SEM, except for those in scatter plots (mean). When comparing multiple conditions, one-way analysis of variance (ANOVA) followed by Bonferroni’s test was performed to identify which groups showed significant differences.

## Results

### Protein extraction from FFPE sections of mouse liver by PCT treatment

**Figure 1:**
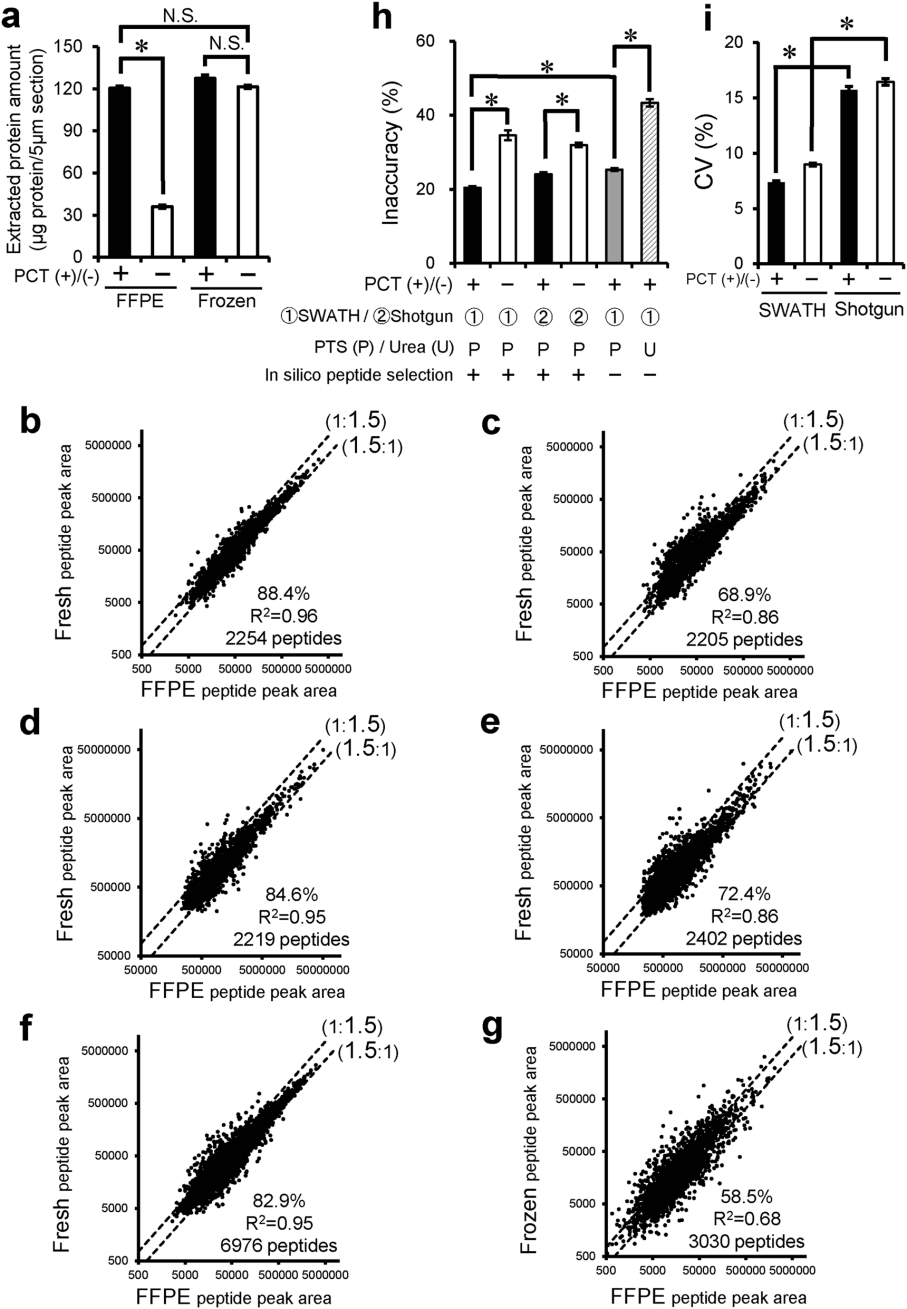
Effect of PCT treatment, SWATH analysis, heat-compatible PTS buffer and in silico peptide selection criteria on comprehensive quantitative proteomics using FFPE sections of mouse liver. (**a**) Protein was extracted from FFPE and frozen sections of matched mouse liver with or without PCT. The experimental procedure is described in “Materials and methods”. Each column represents the mean ± SEM (n = 4). *p < 0.001, significant difference between two groups. N.S., no significant difference between two groups (*p* > 0.001) (Bonferroni-corrected Student’s *t*-test). (**b**–**f**) Peptide peak areas were compared between FFPE and fresh mouse livers. Peptide samples of FFPE and fresh livers were prepared with PCT treatment using PTS buffer (95 °C heating for lysis) and measured in the SWATH mode [**b**,**f**, PCT(+)-SWATH], prepared without PCT treatment (only incubation in PTS buffer at 95 °C for lysis) and measured in the SWATH mode [**c**, PCT(−)-SWATH], prepared with PCT treatment using PTS buffer (95 °C heating for lysis) and measured in the shotgun mode [**d**, PCT(+)-Shotgun], or prepared without PCT treatment (only incubation in PTS buffer at 95 °C for lysis) and measured in the shotgun mode [**e**, PCT(−)-Shotgun]. Data analysis for (**b**–**e**) was carried out as shown in Supplementary Fig. 2 with in silico peptide selection criteria. The data were taken from Supplementary Tables 2–5. Data analysis for f was carried out as shown in Supplementary Fig. 2, but without application of the in silico peptide selection criteria. Each point represents the mean (n = 4). The broken lines represent 1.5-fold differences. The % in each scatter plot is the proportion of peptides whose peak areas from FFPE samples lie within a 1.5-fold range of those from fresh samples. (**g**) This graph was generated using the data from the reported PCT-SWATH study.^7^ Peptide peak areas were compared between FFPE and frozen human benign prostatic tissues obtained from the same resected tissue of patient 15, who showed the best agreement (as an inaccuracy value) between FFPE and frozen peptide peak areas among the 24 patients. The peptide samples of FFPE and frozen prostatic tissues were prepared with PCT treatment using urea buffer without heating, and then measured in the SWATH mode. The in silico peptide selection criteria were not applied. The broken lines represent 1.5-fold differences. The % in each scatter plot is the proportion of peptides whose peak areas from FFPE samples lie within a 1.5-fold range of those from frozen samples. (**h**,**i**) Inaccuracy (**h**) and CV values (**i**) were calculated from panels b, c, d and e as described in “Materials and methods”, and the inaccuracy (**h**) were also calculated from panels (**f**) (gray column) and (**g**) (hatched column). Each column represents the mean ± SEM (n = 2,205–6,976 peptides; number commonly detected in FFPE and fresh samples under each experimental condition). *p < 0.001, significant difference between two groups (Bonferroni-corrected Student’s t-test).

To clarify the efficiency of extraction by PCT treatment from FFPE sections, we compared the protein amounts extracted from FFPE and frozen sections. It was assumed that proteins in frozen sections are efficiently extracted. This assumption is supported by the observation that the extracted protein amount from frozen sections was not increased by the addition of PCT treatment (Fig. 1a). In the case of FFPE sections, PCT treatment (PCT(+)) significantly increased the extracted protein amount by 3.33-fold compared to no PCT treatment (PCT(−)), and the amount extracted reached the same level as that from frozen sections (Fig. 1a). These results suggest that our protocol can extract protein from FFPE sections with almost 100% efficiency.

**Figure 2:**
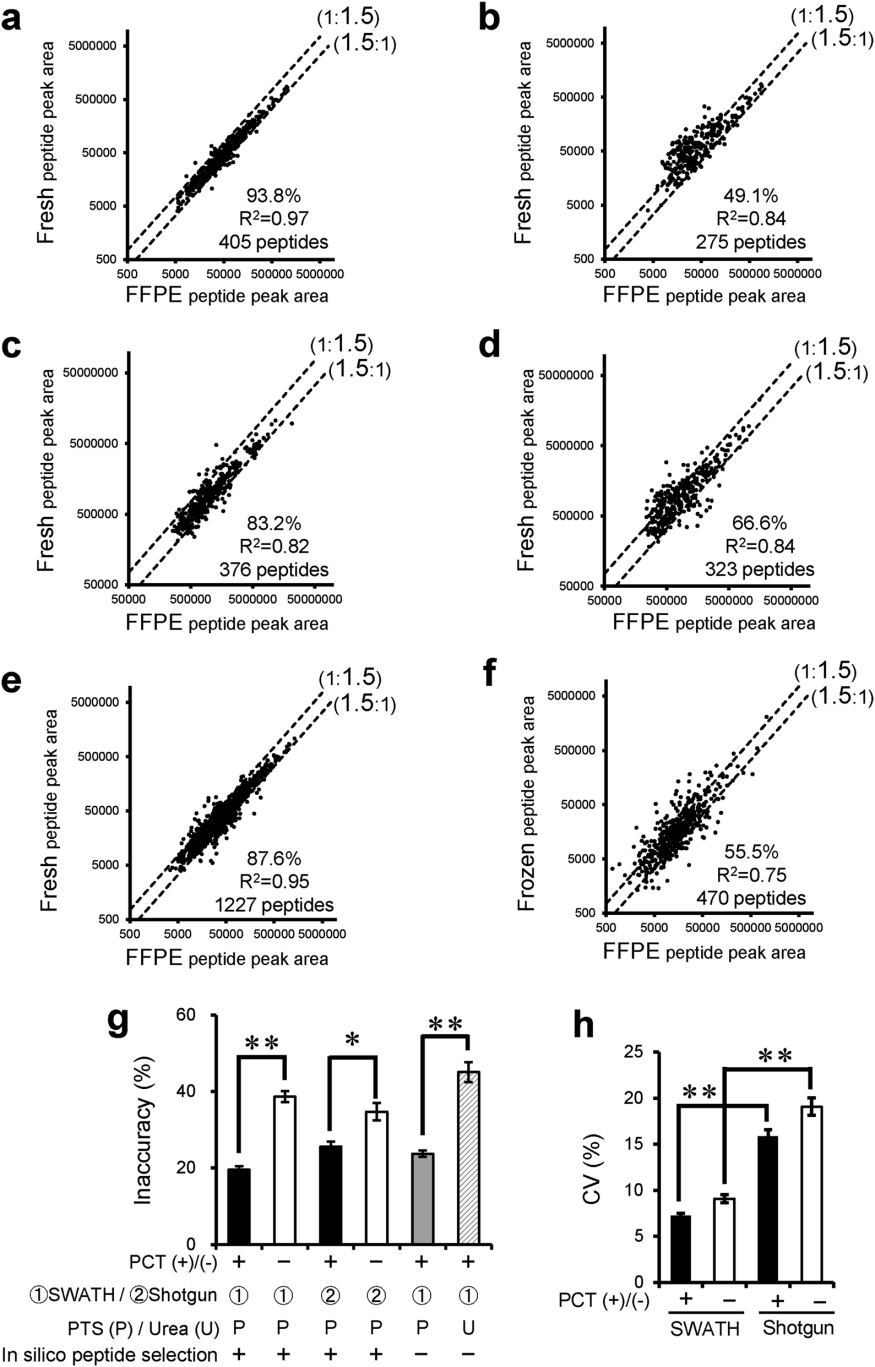
Effect of PCT treatment, SWATH analysis, heat-compatible PTS buffer and in silico peptide selection criteria on comprehensive quantification of membrane proteins using FFPE sections of mouse liver. (**a–e**) Membrane proteins were selected according to the subcellular location information in the Uniprot mouse proteome database. Peptide peak areas were compared between FFPE and fresh mouse livers. Peptide samples of FFPE and fresh livers were prepared with PCT treatment using PTS buffer (95 °C heating for lysis) and measured in the SWATH mode (**a**, **e**, PCT(+)-SWATH], prepared without PCT treatment (only incubation in PTS buffer at 95 °C for lysis) and measured in the SWATH mode [**b**, PCT(-)-SWATH], prepared with PCT treatment using PTS buffer (95 °C heating for lysis) and measured in the shotgun mode [**c**, PCT(+)-Shotgun], or prepared without PCT treatment (only incubation in PTS buffer at 95 °C for lysis) and measured in the shotgun mode [**d**, PCT(-)-Shotgun]. Data analysis for (**a**–**d**) was carried out as described in Supplementary Fig. 2 with in silico peptide selection criteria. The data were taken from Supplementary Tables 2–5. Data analysis for e was carried out as shown in Supplementary Fig. 2 but without application of the in silico peptide selection criteria. Each point represents the mean (n = 4). The broken lines represent 1.5-fold differences. The % in each scatter plot is the proportion of peptides whose peak areas from FFPE samples lie within a 1.5-fold range of those from fresh samples. (**f**) This graph was generated using the data from the reported PCT-SWATH study.^7^ Membrane proteins were selected according to the subcellular location information in the Uniprot human proteome database. Peptide peak areas were compared between FFPE and frozen human benign prostatic tissues obtained from the same resected tissue of patient 15, who showed the best agreement (as an inaccuracy value) between FFPE and frozen peptide peak areas among the 24 patients. The peptide samples of FFPE and frozen prostatic tissues were prepared with PCT treatment using urea buffer without heating, and then measured in the SWATH mode. The in silico peptide selection criteria were not applied in this case. The broken lines represent 1.5-fold differences. The % in each scatter plot is the proportion of peptides whose peak areas from FFPE samples lie within a 1.5-fold range of those from frozen samples. (**g**,**h**) Inaccuracy (**g**) and CV values (**h**) were calculated from panels (**a**–**d**) as described in “Materials and methods”, and the inaccuracy (**g**) were also calculated from panels (**e**) (gray column) and (**f**) (hatched column). Each column represents the mean ± SEM (n = 275–1,227 peptides; number commonly detected in FFPE and fresh samples under each experimental condition). **p < 0.001, *p < 0.01, significant difference between two groups (Bonferroni-corrected Student’s t-test).

### Effect of PCT treatment, SWATH analysis, heat-compatible PTS buffer and in silico peptide selection criteria on comprehensive protein quantification in FFPE sections of mouse liver

To clarify whether PCT-assisted sample processing and SWATH analysis enable reproducible protein quantification from FFPE samples and give the same protein expression levels as found in fresh tissue, we compared this protocol with the conventional method (without PCT (PCT(−)); shotgun analysis). This comparison was made in terms of how well the peak areas of tryptic peptides (those detected from both FFPE and fresh samples) obtained from FFPE sections agree with those obtained from fresh tissues, and how reproducible they are (Fig. 1 and Supplementary Tables 2–5). When PCT treatment was used, the percentage of peptides whose peak areas lie within a 1.5-fold range of those in fresh samples was significantly increased from 68.9% (Fig. 1c, PCT(−)) to 88.4% (Fig. 1b, PCT(+)), and the inaccuracy was significantly decreased from 34.6 to 20.4% (Fig. 1h). In addition, SWATH significantly improved the CV values of quantification, a parameter describing the variability of the peak areas of peptides among 4 replicates, in FFPE sections from 15.7 to 7.39% (Fig. 1i).

The present method (PCT(+)-SWATH) was further compared to the conventional PCT-SWATH method^7^, which differs in two respects, as follows. (1) The conventional method uses a urea lysis buffer, while the present one employs the PTS buffer and 95 °C heating for decrosslinking and protein extraction. (2) The conventional method does not employ in silico peptide selection criteria (Supplementary Fig. 2), while the present one does. The previous study using the conventional PCT-SWATH method^7^ compared the quantitative results between FFPE and frozen samples of the same resected prostatic tissues from 24 donors. Because the FFPE and frozen tissue samples were taken from adjacent regions of the same resected prostatic tissue, it was assumed that the true protein expression profiles would be the same in the FFPE and frozen samples. Because a large interindividual difference was observed in the degree of agreement between FFPE and frozen peptide peak areas, the benign prostatic tissue of patient 15, who showed the best agreement (the smallest inaccuracy among the 24 donors), was selected and the FFPE peptide peak areas were compared with the frozen peptide peak areas. (Note that we did not conduct any experiment using human tissues in the present study. We just took the raw data of human proteome analysis from a previous study and used it for our data analysis). The percentage of peptides within a 1.5-fold range was 58.5% (Fig. 1g), which is very much smaller than that (88.4%, Fig. 1b) obtained under the PCT(+)-SWATH condition established in the present study. The inaccuracy under the PCT(+)-SWATH condition without applying in silico peptide selection criteria (i.e., the urea buffer without heating in the conventional PCT-SWATH method was changed to PTS buffer with heating) was significantly smaller (25.3%) than that (43.4%) of the conventional PCT-SWATH method (Fig. 1h). The inaccuracy was further significantly decreased from 25.3 to 20.4% by applying the in silico peptide selection criteria as well (Fig. 1h). These results suggest that (1) the use of PTS buffer and 95 °C heating in the step of decrosslinking and protein extraction and (2) the in silico peptide selection criteria are both useful to obtain quantitative results that well reflect those obtained with fresh tissue.

To examine whether PCT treatment is also effective for membrane proteins in FFPE sections, the same analysis as above was separately conducted for membrane and cytosol proteins. Cytosolic and membrane proteins were selected according to the subcellular location information in the Uniprot mouse or human proteome database. Among cytosol proteins, the percentage of tryptic peptides whose peak areas lie within a 1.5-fold range of those in fresh samples was 73.2% without PCT treatment (PCT(−)) and 85.5% with PCT treatment (PCT(+))(Supplementary Fig. 4a and b). Among membrane proteins, the corresponding values were 49.1% (Fig. 2b, PCT(−)) and 93.8% (Fig. 2a, PCT(+)), demonstrating a dramatic increase upon PCT treatment. Furthermore, PCT increased the number of peptides detected by 1.47-fold (405 peptides in Fig. 2a divided by 275 peptides in Fig. 2b). These data suggest that PCT is especially effective in promoting the de-crosslinking, extraction and digestion of membrane proteins.

For both cytosolic and membrane proteins, the inaccuracy under the conventional PCT-SWATH condition using urea buffer (without peptide selection criteria) was significantly larger than that of PCT(+)-SWATH using PTS buffer without peptide selection criteria (Fig. 2g and Supplementary Fig. 4g). This suggests that the use of PTS buffer and 95 °C heating in the step of decrosslinking and protein extraction is useful for both cytosolic and membrane proteins.

### Effect of PCT treatment on the qualitative protein detection profile from FFPE sections of mouse liver

**Figure 3:**
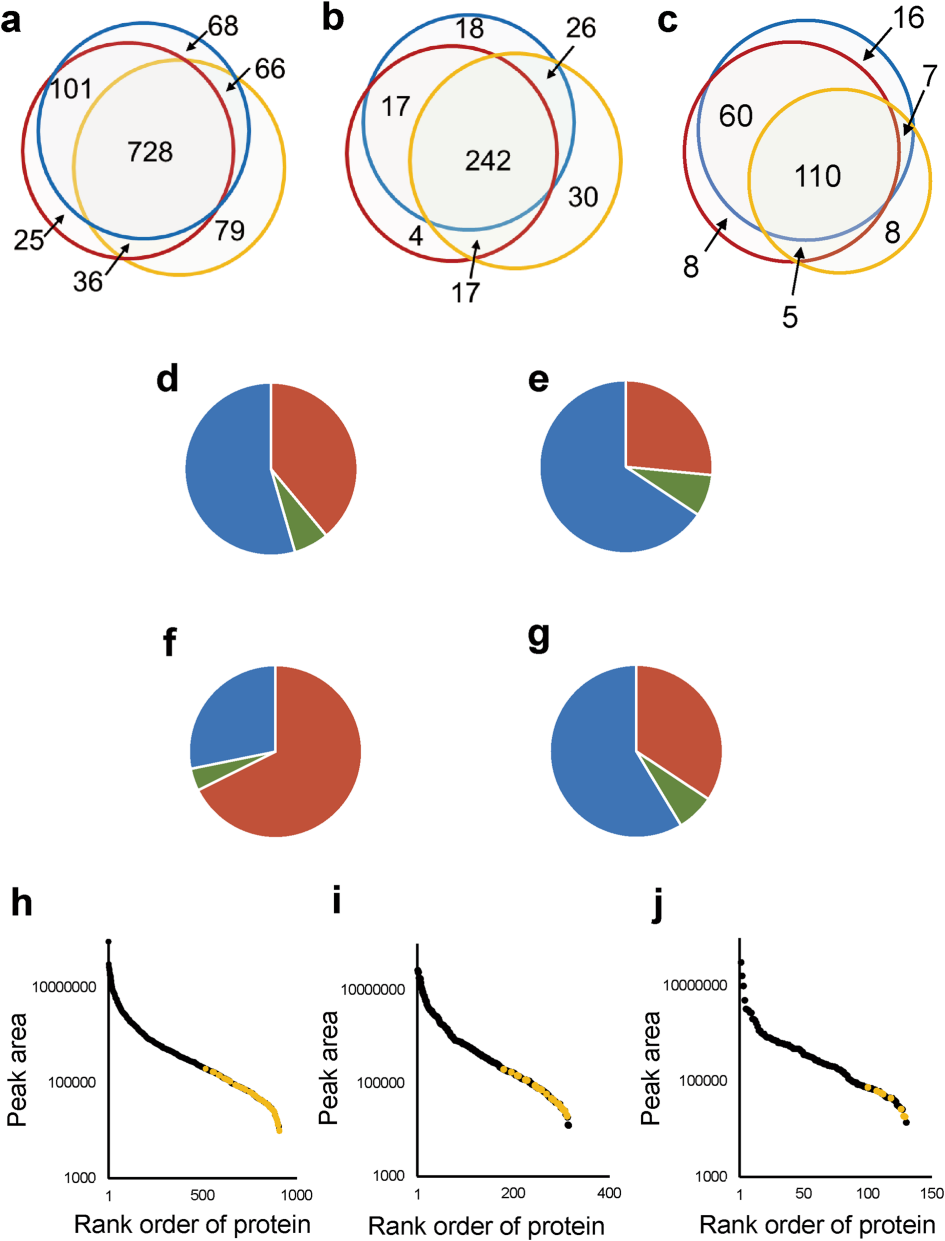
Effect of PCT treatment on qualitative protein detection profile in FFPE sections of mouse liver. (**a**–**c**) The overlap of proteins detected from FFPE samples (red circle, PCT(+)-SWATH; yellow circle, PCT(−)-SWATH) with those detected from fresh samples (blue, PCT(+)-SWATH) is shown in Venn diagrams for total (**a**), cytosolic (**b**) and membrane (**c**) proteins. The number of proteins is shown in each region of the Venn diagrams. The subcellular localization was taken from the Uniprot mouse proteome database. (**d**–**g**) For total (**d**), cytosolic (**e**), membrane (**f**), and non-membrane (**g**) proteins, the changes in peak area caused by PCT treatment in FFPE samples were investigated using the data obtained under the conditions of PCT(+)-SWATH and PCT(−)-SWATH (Supplementary Tables 2 and 3). Red, increase by more than 1,000 counts. Blue, decrease by more than 1,000 counts. Green, change of no more than 1,000 counts. (**h**–**j**) The peak areas of all proteins detected under the PCT(−)-SWATH condition of FFPE sample were plotted for total (**h**), cytosolic (**i**), and membrane (**j**) proteins. Yellow plot, the peak area of proteins only detected under the PCT(−)-SWATH condition from FFPE samples, but not detected from FFPE or fresh samples under the PCT(+)-SWATH condition. The peak area of each protein was calculated as the sum of peak areas of all transitions derived from the corresponding protein.

The above analysis focused only on peptides that were detected in both FFPE and fresh samples. To address the effect of PCT more rigorously, we analyzed the overlap of proteins detected in FFPE and fresh samples by using Venn diagrams for FFPE samples processed with (PCT(+)) or without PCT treatment (PCT(−)) (Fig. 3). The three groups represent all proteins, cytosolic proteins, and membrane proteins (Fig. 3a–c, respectively). The numbers of proteins that overlapped between fresh and FFPE samples with PCT treatment (PCT(+)) (101 + 728 for all proteins; 17 + 242 proteins for cytosolic proteins) were similar to those between fresh samples with PCT treatment (PCT(+)) and FFPE samples without PCT treatment (PCT(−)) (66 + 728 for all proteins; 26 + 242 proteins for cytosolic proteins) (Fig. 3a,b). By contrast, the number of membrane proteins that overlapped between fresh and FFPE samples with PCT treatment (PCT(+)) (60 + 110 proteins) was higher than that between fresh samples with PCT treatment (PCT(+)) and FFPE samples without PCT treatment (PCT(−)) (7 + 110 proteins) (Fig. 3c). The PCT treatment increased the percentage of proteins overlapping between FFPE and fresh samples from 60.6 to 88.1% of the total proteins in fresh sample (Fig. 3c). Compared with the total proteins in fresh samples, those not detected under the PCT(−) condition but detected under the PCT(+) condition in FFPE samples are listed in Supplementary Table 6 [101 proteins (Fig. 3a), including 17 cytosolic (Fig. 3b) and 60 membrane proteins (Fig. 3c)].

For total, cytosolic, membrane and non-membrane proteins (proteins other than membrane proteins), the percentages of proteins whose peak areas were increased or decreased by PCT are shown in pie charts in Fig. 3d–g, respectively. When the SWATH data were compared under the PCT(+) and PCT(−) conditions, the peak areas for 67.7% of membrane proteins were increased by more than 1,000 counts by PCT treatment (Fig. 3f). In contrast, PCT caused decreases of more than 1,000 counts for 54.5%, 65.7%, and 58.6% of total, cytosolic and non-membrane proteins, respectively (Fig. 3d,e,g).

The peak areas of 79, 30 and 8 proteins which were only detected under the PCT(−) condition in the Venn diagrams of total, cytosolic and membrane proteins, respectively (Fig. 3a–c), were relatively small (yellow plots) among all proteins detected under the PCT(−) condition (Fig. 3h–j).

The median numbers of amino acid residues (AAs) of proteins in each region of the Venn diagrams in Fig. 3a–c are shown in Supplementary Fig. 5a–c, respectively. For cytosolic proteins, the median under the PCT(−) condition was 334 AAs. This is small compared to that (548 AAs) of proteins commonly detected in FFPE and fresh samples under the PCT(+) but not the PCT(−) condition (Supplementary Fig. 5b). For membrane proteins, the median under the PCT(−) condition was 462.5 AAs, which is almost the same as that (475 AAs) of proteins commonly detected in FFPE and fresh samples under the PCT(+) but not the PCT(−) condition (Supplementary Fig. 5c). However, the percentage of transmembrane proteins among the membrane proteins only detected in the PCT(−) condition was 62.5%, which is small compared to that (83.3%) of transmembrane proteins among membrane proteins commonly detected in FFPE and fresh samples under the PCT(+) but not the PCT(−) condition (Supplementary Fig. 6).

### Effect of PCT treatment, SWATH analysis, heat-compatible PTS buffer and peptide/data selection criteria on comprehensive quantification of pathological changes in protein expression level in FFPE sections

**Figure 4:**
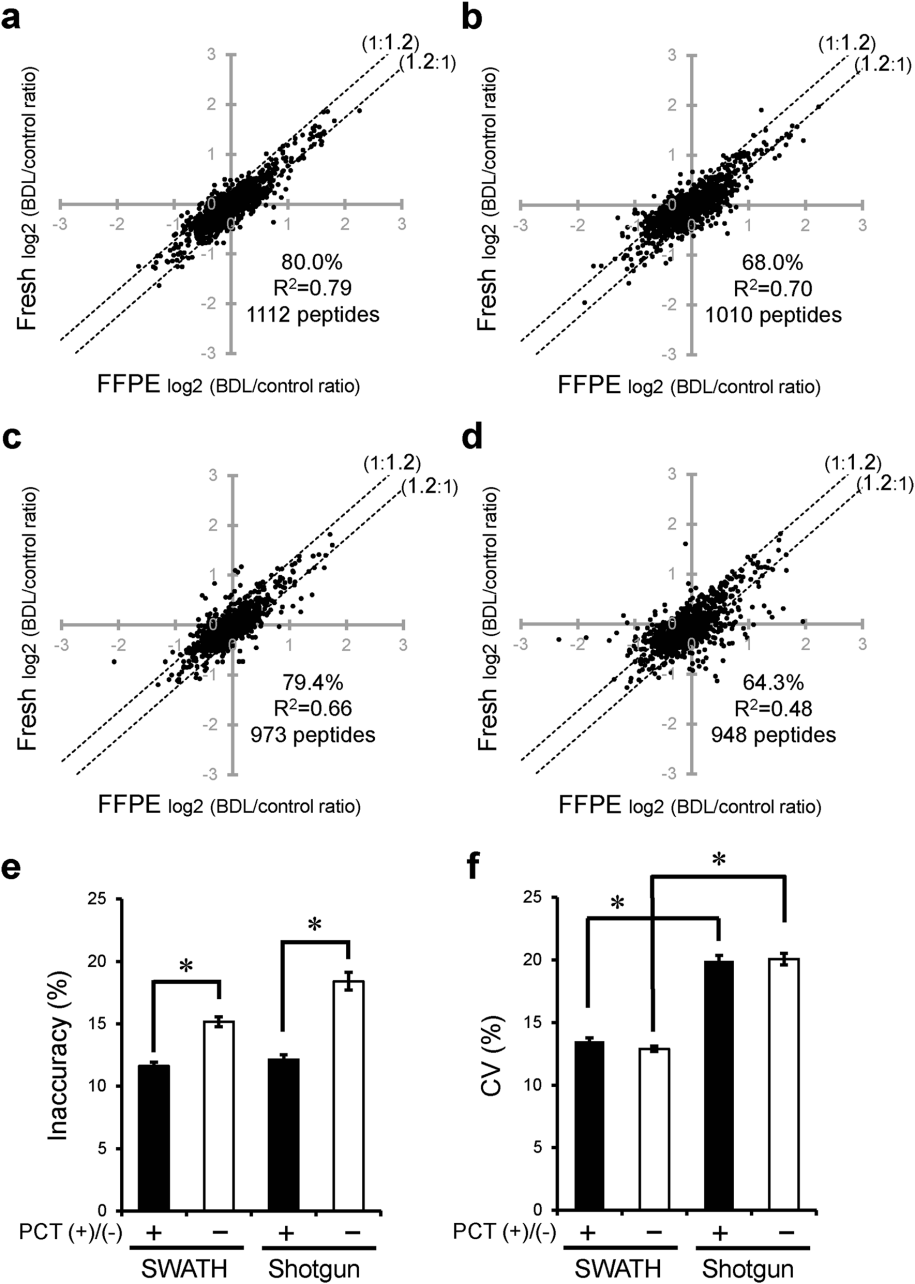
Effect of PCT treatment and SWATH analysis on comprehensive quantification of BDL-induced changes in protein expression using FFPE sections. (**a**–**d**) BDL-induced changes in expression level (BDL/control ratio) were compared between FFPE and fresh samples of mouse liver at the peptide level. Peptide samples of FFPE and fresh livers were prepared with PCT treatment and measured in the SWATH mode (**a**, PCT(+)-SWATH), prepared without PCT treatment and measured in the SWATH mode (**b**, PCT(−)-SWATH), prepared with PCT treatment and measured in the shotgun mode (**c**, PCT(+)-Shotgun), or prepared without PCT treatment and measured in the shotgun mode (**d**, PCT(−)-Shotgun). Data analysis was carried out as described in Supplementary Fig. 3. The data were taken from Supplementary Tables 7–10. Each point represents the mean (n = 4). The broken lines represent 1.2-fold differences. The % in each scatter plot is the proportion of peptides whose peak areas from FFPE samples lie within a 1.2-fold range of those from fresh samples. (**e**,**f**) Inaccuracy (**e**) and CV values (**f**) obtained from panels (**a**–**d**) were calculated as described in “Materials and methods”. Each column represents the mean ± SEM (n = 948–1,112 peptides; number commonly detected from FFPE and fresh samples under each experimental condition). *p < 0.001, significant difference between two groups (Bonferroni-corrected Student’s t-test).

To verify the usefulness of the FFPE-PCT-SWATH proteomics protocol established in the present study for studies of pathological molecular mechanisms and biomarker discovery, we examined whether the pathological changes in protein expression levels quantified in FFPE sections by means of FFPE-PCT-SWATH proteomics quantitatively agree with those in fresh tissue, using BDL mouse liver as a disease model tissue (Fig. 4 and Supplementary Tables 7–10). We also examined whether the changes are reproducible among 4 replicates. After PCT treatment, the percentage of tryptic peptides whose pathological changes in expression level in FFPE samples lay within a 1.2-fold range of those in fresh samples was significantly increased from 68.0 (Fig. 4b) to 80.0% (Fig. 4a), and the inaccuracy was significantly decreased from 15.2 to 11.6% (Fig. 4e). SWATH significantly improved the CV (a parameter describing variability) of the BDL/control ratios of peak areas of peptides among the 4 replicates in FFPE sections from 19.9 to 13.5% (Fig. 4f). Combined use of PCT and SWATH to examine FFPE sections (FFPE-PCT-SWATH proteomics) remarkably improved the percentage (80.0%, Fig. 4a) of peptides whose pathological changes in expression level in FFPE samples were within a 1.2-fold range of those in fresh samples, as compared to the conventional method (64.3%, Fig. 4d).

When we focused on cytosol proteins, FFPE-PCT-SWATH proteomics increased the percentage (77.2%, Supplementary Fig. 7a) of tryptic peptides whose pathological changes in expression level in FFPE samples lay within a 1.2-fold range of those in fresh samples, as compared to the conventional method (65.2%, Supplementary Fig. 7d). It should be noted that no significant difference was observed in the value of inaccuracy in SWATH analysis with and without PCT treatment (Supplementary Fig. 7e). As for membrane proteins, FFPE-PCT-SWATH proteomics increased the percentage (85.8%, Supplementary Fig. 8a) of tryptic peptides whose pathological changes in expression level in FFPE samples lay within a 1.2-fold range of those in fresh samples, as compared to the conventional method (64.7%, Supplementary Fig. 8d). The value of inaccuracy in SWATH analysis was significantly decreased from 15.8% to 9.80% by PCT treatment (Supplementary Fig. 8e). Further, the CV value in the case of PCT treatment was significantly decreased from 17.8% to 12.5% by SWATH analysis (Supplementary Fig. 8f).

The present method (PCT(+)-SWATH) is further compared to the conventional PCT-SWATH method^7^ using urea buffer and without peptide/data selection in Supplementary Figs. 2 and 3. In the previous study using the conventional PCT-SWATH method^7^, not only benign but also tumorous prostatic tissues from 24 donors were analyzed. Because the FFPE and frozen samples were obtained from same resected prostatic tissues, it was assumed that the pathological changes (tumor/benign ratios) in peptide peak areas would be the same in FFPE and frozen samples. Because a large interindividual difference was observed in the degree of agreement between FFPE and frozen tumor/benign ratios, patient 15 who showed the best agreement (the smallest inaccuracy among the 24 donors) was selected, and the FFPE tumor/benign ratios were compared with the frozen tumor/benign ratios in patient 15. The percentage of peptides within a 1.2-fold range was 28.3% (Supplementary Fig. 9c), which is remarkably smaller than that (80.0%, Supplementary Fig. 9a) obtained under the PCT(+)-SWATH condition established in the present study. Even under the PCT(+)-SWATH condition using PTS buffer but without the in silico peptide selection of Supplementary Fig. 2 and data selection of Supplementary Fig. 3, the percentage of peptides within a 1.2-fold range was 57.7% (Supplementary Fig. 9b). Also, the inaccuracy (21.2%, Supplementary Fig. 9d) was significantly smaller than that of the conventional PCT-SWATH method using urea buffer (51.0%, Supplementary Fig. 9d). These results suggest that the use of PTS buffer and 95 °C heating in the step of decrosslinking and protein extraction is useful to obtain quantitative data that accurately reflect the pathological changes in the fresh tissue.

For both cytosolic and membrane proteins, the inaccuracy under the conventional PCT-SWATH condition using urea buffer without peptide/data selection criteria was significantly larger than that of PCT(+)-SWATH using PTS buffer without peptide/data selection criteria (Supplementary Figs. 10d and 11d). For membrane proteins, the inaccuracy was further significantly decreased by applying the peptide/data selection (Supplementary Figs. 11d). These results suggest that not only the use of PTS buffer and 95 °C heating in the step of decrosslinking and protein extraction, but also the application of peptide/data selection is useful for membrane proteins.

Note that the term peptide/data selection indicates (1) the selection of reliable peptides among all the detected peptides in terms of quantitative performance based on the in silico peptide selection criteria described in Supplementary Fig. 2 and (2) the removal of unreliable peak area data such as unstable peak area among replicates and peak areas that are too small in relation to the background noise level as described in Supplementary Figs. 2 and 3. In other words, this peptide/data selection protocol picks up reliable peptides and peak areas among all the detected peptides and peak areas, in order to ensure that valid comparisons can be made among the experimental conditions.

### Verification of the present FFPE-PCT-SWATH method for the quantification of physiologically and pharmacologically essential pathways and proteins

**Figure 5:**
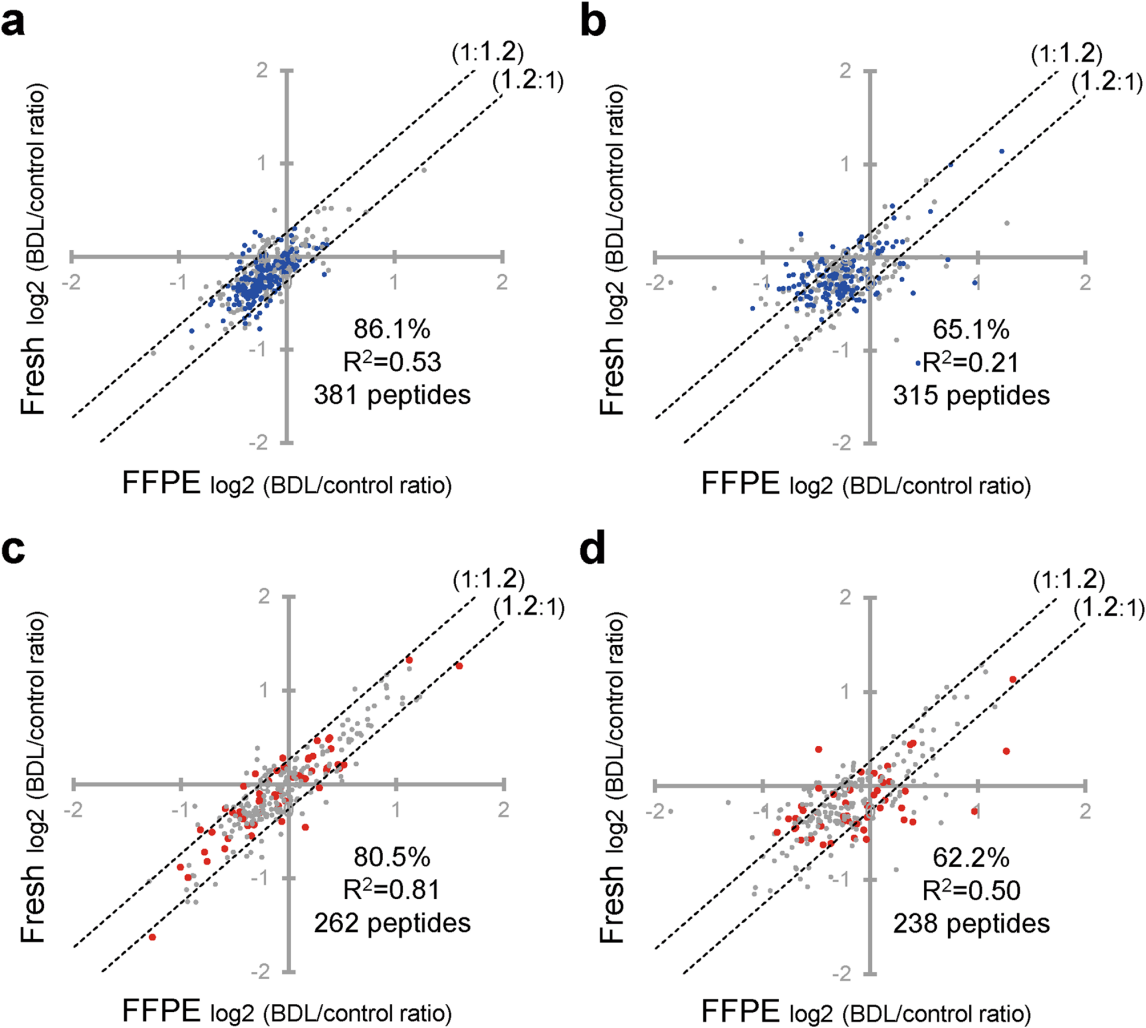
Validation of the present FFPE-PCT-SWATH method for the quantification of BDL-induced changes in protein expression levels of mitochondrial proteins and drug-interacting proteins. The BDL/control ratios were compared between FFPE and fresh samples of mouse liver at the peptide level. Peptide samples of FFPE and fresh livers were prepared with PCT treatment and measured in the SWATH mode (a and c, PCT(+)-SWATH), or prepared without PCT treatment and measured in the shotgun mode (**b**,**d**, PCT(−)-Shotgun). Data analysis was carried out as described in Supplementary Fig. 3. The data were taken from Supplementary Tables 11–14. Each point represents the mean (n = 4). The broken lines represent 1.2-fold differences. The % in each scatter plot is the proportion of peptides whose peak areas from the FFPE samples lie within a 1.2-fold range of those from fresh samples. (**a**,**b**) The mitochondrial proteins were extracted based on Uniprot proteome database and their BDL/control ratios were plotted. The proteins annotated as belonging to the TCA cycle, respiratory electron transport, fatty acid beta-oxidation, and mitochondrial calcium ion transport were selected based on Reactome and Uniprot proteome databases, and are shown in blue. (**c**,**d**) Proteins (enzymes, transporters, receptors, etc.) that would potentially interact with drugs and chemicals were extracted based on the database available freely on the IUPHAR/BPS Guide to Pharmacology website. Proteins belonging to the Cyp, Ugt, Aldh, Adh, Comt, Sult, Ces, Fmo, Tpmt, Acsm, Abc, Slc, and Lrp families were additionally extracted because some of these drug-associated proteins were not listed in the above database. Proteins detected only under either the PCT(+)-SWATH or PCT(−)-Shotgun condition are shown in red.

To verify the usefulness of our established FFPE-PCT-SWATH method for quantifying pathological changes of physiologically essential pathways, we extracted the BDL/control ratios for mitochondrial proteins. We focused on ATP-related pathways (blue in the figures and tables), such as respiratory electron transport, TCA cycle, and fatty acid beta-oxidation (Fig. 5 and Supplementary Tables 11 and 12), which have been reported to be impaired in BDL liver^16,17^. By applying PCT and SWATH, the percentage of tryptic peptides whose pathological changes in expression level in FFPE samples lay within a 1.2-fold range of those in fresh samples was increased from 65.1% (Fig. 5b, PCT(−)-Shotgun) to 86.1% (Fig. 5a, PCT(+)-SWATH). As shown in the blue plots, the BDL-induced decreases in the expression levels of ATP synthesis-related systems in FFPE samples quantitatively agreed well with those observed in fresh tissues under the PCT(+)-SWATH condition, as compared to the PCT(−)-Shotgun condition.

We also extracted proteins (enzymes, transporters, receptors, etc.) that could potentially interact with drugs and chemicals, by using the database available freely on the IUPHAR/BPS Guide to Pharmacology website (https://www.guidetopharmacology.org/download.jsp). Proteins belonging to the Cyp, Ugt, Aldh, Adh, Comt, Sult, Ces, Fmo, Tpmt, Acsm, Abc, Slc, and Lrp families were additionally extracted, because some of these drug-associated proteins were not listed in the above database. As shown in Fig. 5c and d (Supplementary Tables 13 and 14), the BDL/control ratios in FFPE samples agreed well with those in fresh samples under the PCT(+)-SWATH condition, as compared to the PCT(−)-Shotgun condition. As shown in the red plots, most of the proteins that were only detected under the PCT(+)-SWATH condition were identical within a 1.2-fold range in FFPE and fresh samples (Fig. 5c).

These protein names and data are listed in Supplementary Tables 11–14.

## Discussion

Here, we have established a significantly improved quantitative proteomics protocol (highly accurate FFPE-PCT-SWATH) that is able to accurately and comprehensively quantify the proteins, including membrane proteins, and pathological changes of protein expression levels in FFPE samples. This method provides quantitative results that reflect the protein expression levels in fresh tissue much more accurately than does conventional PCT(−)-Shotgun-based FFPE proteomics (Fig. 1), conducted using a protocol similar to that reported previously^13^. A previous study^7^ employed the conventional PCT-SWATH protocol, which differs from the present study in using urea buffer without heating and in not employing in silico peptide selection criteria. In that study, only 58.5% of peptides agreed within a 1.5-fold range between FFPE and frozen tissues in terms of peptide peak area (Fig. 1g). In contrast, 88.4% of peptides agreed in the case of the FFPE-PCT-SWATH method established in the present study (Fig. 1b). Although the CV value of FFPE samples was 27.7% in the previous study^7^, it was 7.39% in the present FFPE-PCT-SWATH method (Fig. 1i). The main reason for these significant improvements is considered to be that (1) PTS buffer is superior to urea buffer for protein extraction from FFPE section^10^, (2) the decrosslinking and protein extraction are also promoted by heating at 95 °C, which is incompatible with the use of urea buffer, and (3) the in silico peptide selection criteria are useful to remove quantitatively inappropriate peptides. This new methodology has also enabled accurate and comprehensive quantification of the pathological changes in protein expression levels in a disease model from FFPE sections for the first time. Importantly, it provides results in good agreement with those obtained from fresh tissues (Fig. 4) not only for soluble proteins, but also for membrane proteins (Supplementary Figs. 7 and 8). We consider that the use of PCT, PTS buffer and 95 °C heating contributed to the good agreement between the pathological changes of protein expression levels found in FFPE and fresh samples (Fig. 4, and Supplementary Figs. 7–11).

We showed that the use of PCT significantly improved the decrosslinking and extraction of proteins from FFPE sections (Fig. 1a), thus overcoming the most critical issue in FFPE proteomics. This dramatically increased the accuracy of comprehensive quantification (i.e., the similarity of the quantification results in FFPE samples to those in fresh ones) (Figs. 1 and 4). PCT was especially effective for membrane proteins (Fig. 2 and Supplementary Fig. 8). Without PCT treatment, 90.0% of all the peptides whose peak areas did not lie within a 1.5-fold range in FFPE and fresh samples showed a > 1.5-fold smaller peak area in FFPE samples (Fig. 2b). Almost all of these peptides showed an increased peak area after PCT treatment, resulting in good agreement within a 1.5-fold range (Fig. 2a). As shown in Fig. 3c, the percentage of membrane proteins commonly detected in FFPE and fresh samples was 60.6% without PCT treatment, but was dramatically increased to 88.1% with PCT treatment. These results indicate that the decrosslinking, extraction and LysC/trypsin digestion of membrane proteins were incomplete in the conventional method, but were dramatically improved by PCT treatment. A possible explanation could be that the alternating cycles of hydrostatic high pressure and ambient pressure by PCT accelerate the hydrolysis of formalin crosslinking (methylene bridge) of proteins in FFPE sections and the dissociation of protein-lipid bilayer interaction, leading to efficient solubilization of membrane proteins. The heating at 95 °C could be an important factor to facilitate these reactions, and in addition PTS (heat compatible) solubilizes the membrane proteins more efficiently than urea. Furthermore, PCT and PTS could both facilitate the denaturation of protein substrates, which would favor efficient digestion by LysC/trypsin.

Furthermore, the variability of quantitative values among four independent sample preparations was significantly smaller in SWATH than in shotgun analysis (Figs. 1 and 4), suggesting that SWATH increased the reproducibility. The CV values were decreased to less than 10% by using SWATH (Fig. 1i). This high precision is comparable to that of targeted quantitative proteomics including SRM analysis^18^. A previous study showed a CV value of 12.1% in SRM quantification for FFPE specimens^4^, so the present SWATH analysis of FFPE specimens is slightly superior in terms of reproducibility, notwithstanding the comprehensive quantification. Overall, our results suggest that it is essential to combine PCT and SWATH in order to achieve accurate and reproducible comprehensive protein quantification from FFPE sections.

Although pathological changes in protein expression levels have been measured by proteomics analysis using FFPE specimens, it is not clear whether they quantitatively reflect the in vivo pathological changes. In a quantitative comparison between FFPE and frozen tissues of human renal cell carcinoma (Rapigest protocol with a high-resolution mass spectrometry system), the tumor/normal expression ratios of proteins were poorly correlated between FFPE and frozen tissues (Pearson R^2^ = 0.47)^19^. Here, we obtained a Pearson R^2^ of 0.48 under the PCT(−)-Shotgun condition, but this was significantly increased to 0.79 by combining PCT and SWATH (Fig. 4). In particular, a large improvement of Pearson R^2^ was observed for membrane proteins under the PCT(+)-SWATH condition (Pearson R^2^ = 0.73), as compared with the PCT(−)-Shotgun condition (Pearson R^2^ = 0.29) (Supplementary Fig. 8. Furthermore, under the PCT( +)-SWATH condition for membrane proteins, 85.8% of peptides showed BDL/control ratios within a 1.2-fold range between fresh and FFPE samples (Supplementary Fig. 8a). Although the above data were obtained after peptide/data selection (Supplementary Figs. 2 and 3) was applied, we found that even if this selection was omitted, the PCT-SWATH method using the PTS buffer with heating (Supplementary Figs. 9b, 10b and 11b) gave quantitative results that reflect the pathological changes in protein expression levels in fresh tissue much more accurately than does the conventional PCT-SWATH method using urea buffer (Supplementary Figs. 9c, 10c and 11c). These findings suggest that our established FFPE-PCT-SWATH method using PTS buffer and 95 °C heating can accurately determine the pathological changes in expression levels for proteins including membrane proteins.

In the present study, we compared the data generated by our present method and the published PCT-SWATH method in order to assess the superiority of our method. However, it must be noted that this comparison has some serious limitations, because different tissue samples were used to acquire the two datasets (human tumor tissue vs mouse liver). There are also some differences in the methods of LC–MS measurement and SWATH data analysis. Furthermore, substantially different numbers of proteins and peptides were quantified in the two data sets. To properly demonstrate the superiority of the present method, it will be necessary to compare the two methods using the same tissue samples and data analysis protocols.

PCT is also helpful to quantify changes in important molecular mechanisms that are inaccessible to conventional FFPE proteomics. For membrane proteins, the percentage of proteins commonly detected in fresh and FFPE samples was 60.6% under the PCT(−) condition, but this increased to 88.1% with PCT treatment (Fig. 3c). Steroid/lipid metabolism and drug metabolism are two major functions of the liver, and multiple enzymes in these metabolic systems, such as hydroxysteroid dehydrogenase and cyp3a11, were not detected under the PCT(−) condition, but were detected under the PCT(+) condition (Supplementary Table 6). This suggests that it is necessary to use PCT in order to quantify proteins associated with physiologically and pharmacokinetically important molecular mechanisms from FFPE tissues.

Mitochondrial ATP synthesis is impaired in the BDL liver^16,17^. In accordance with those reports, decreased expression levels of ATP synthesis-associated proteins were confirmed in BDL liver (Fig. 5a). Compared to the conventional method (PCT(−)-Shotgun condition, Fig. 5b), the PCT(+)-SWATH condition revealed decreased BDL/control ratios for ATP-related proteins in FFPE samples that agreed well with those observed in fresh samples (Fig. 5a). This result suggests that the FFPE-PCT-SWATH protocol is suitable to investigate pathological changes in molecular mechanisms.

In drug discovery and development, it is important to clarify the molecular mechanisms of drug-induced hepatotoxicity and to identify their biomarkers in order to predict the potential hepatotoxicity of candidate drugs in human clinical trials. To understand whether FFPE specimens are useful for these purposes, we examined whether changes in expression levels of proteins that would potentially interact with drugs and chemicals, such as enzymes, transporters, receptors, etc., can be accurately quantified by FFPE-PCT-SWATH (Fig. 5). The BDL/control ratios in FFPE samples lay within a 1.2-fold range of those in fresh samples for 80.5% of peptides (Fig. 5c), providing a much better performance than the conventional method (PCT(−)-Shotgun condition) (Fig. 5d). A huge number of FFPE tissue samples have been collected and are stored in pharmaceutical and chemical toxicological safety laboratories. Therefore, the FFPE-PCT-SWATH method is expected to find widespread application, and should greatly accelerate studies of the mechanisms and biomarkers of drug-induced hepatotoxicity.

Some proteins, such as 79 proteins in Fig. 3a and 30 proteins in Fig. 3b, were detected in FFPE samples under the PCT(−)-SWATH condition but not under the PCT(+)-SWATH condition for FFPE or fresh samples. This reason for this may be as follows: although protein extraction from FFPE sections under the PCT(−) condition was 3.33-fold smaller than that under the PCT(+) condition (Fig. 1a), the peptide amounts applied to the LC–MS/MS run were adjusted to 1 μg peptide in common for all experimental conditions, in order to evaluate the differences in measured protein expression profile and quantitative performance between PCT( +) and (−) conditions independently of the peptide injection amount. PCT treatment increases the proportions per μg peptide of the tryptic peptides from proteins whose decrosslinking, extraction and LysC/trypsin digestion are promoted. Therefore, the proportions of peptides unaffected by PCT are decreased, so that these peptides show decreased peak intensities in SWATH measurement when the common amount of 1 μg peptide is used for the LC–MS/MS run, resulting in failure of detection. PCT is especially effective for membrane proteins, so non-membrane proteins may be more likely not to be detected under the PCT(+) condition, even if they are detected under the PCT(-) condition. Indeed, the peak areas of more than half of membrane proteins were increased by PCT treatment (Fig. 3f), while the peak areas of more than half of cytosolic proteins were decreased by PCT treatment (Fig. 3e). The 30 cytosolic proteins in Fig. 3b showed small peak areas among all the proteins detected under the PCT(−) condition (Fig. 3i). Thus, it is reasonable that these 30 cytosolic proteins would not be detected because of the decreased peak area after PCT treatment.

Similar considerations apply to the 79 proteins (Fig. 3a) that were not detected under the PCT(+) condition. 71 out of the 79 proteins are non-membrane proteins (Fig. 3a,c). As shown in Fig. 3g, the peak areas of more than half of non-membrane proteins were decreased by PCT treatment. As shown in Fig. 3h, the 79 proteins showed small peak areas among all the proteins detected under the PCT(−) condition. This may explain why these proteins were only detected under the PCT(−) condition in Fig. 3a.

It has been reported that low-molecular-weight and hydrophilic proteins are more likely to be identified in FFPE tissue samples than in frozen samples in the conventional proteomic method^20^. In accordance with this, the median number of AAs (334 AAs) of cytosolic proteins only detected under the PCT(−) condition was smaller than that (548 AAs) of cytosolic proteins commonly detected under the PCT( +) condition in FFPE and fresh samples (Supplementary Fig. 5b). This suggests that PCT is effective to detect high-molecular-weight cytosolic proteins, and may explain why PCT treatment increased the peak areas of even cytosolic proteins (Fig. 3e). In contrast, the effect of PCT treatment on membrane proteins could not be explained in terms of molecular weight (Supplementary Fig. 5c). However, the percentage of proteins with a transmembrane region among the proteins detected only under the PCT(+) condition but not detected in PCT(−) condition (83.3% and 100%, Supplementary Fig. 6) was larger than that among the proteins detected only under the PCT(−) condition (62.5%, Supplementary Fig. 6). This suggests that the PCT may especially promote the preparation of membrane proteins having a transmembrane region and therefore the peak areas of membrane proteins not having a transmembrane region may be relatively decreased by PCT treatment. This would explain why there were proteins whose peak area was decreased by PCT treatment even among membrane proteins (Fig. 3f), and why there were 8 membrane proteins only detected under the PCT(−) condition (Fig. 3c).

In the present study, the data are presented at the peptide level, rather than the protein level, because we expect that data at the peptide level would be more sensitive to changes in quantitative performance arising from changes in experimental conditions. For example, by applying the in silico peptide selection criteria to the SWATH data of control mouse liver processed under the PCT(+) condition using PTS buffer, the inaccuracy was statistically significantly decreased at the peptide level (Fig. 1h), though not at the protein level (from 19.5 to 18.9%, Supplementary Fig. 12e (peak area in control mouse liver)).

The peptide/data selection decreased the number of proteins in the step of data analysis, and the number of proteins detected was only 1,161 without the peptide/data selection (Supplementary Fig. 12). This is considerably smaller than the number of proteins (3,191) quantified in the previous SWATH study using mouse liver^21^, but the main reason for this is likely that the number of proteins quantified by SWATH analysis depends on the number included in spectral library. Our library (3,140 proteins) was about half the size of that (5,935 proteins) used in the previous SWATH study^21^. This is not an issue for the present purpose, since our study is focused on accuracy, but a larger library size would be necessary to realize comprehensive and accurate proteome analysis. However, it should be noted that when more peptides and proteins are quantified, the reproducibility values could likely change, since lower abundance analytes are expected to be more variable.

It is also important to consider the time required for experiments. At maximum, 16 samples can be simultaneously handled in one experiment [the maximum number which can be processed in the PCT machine (Barocycler, NEP 2,320 Enhanced)]. It takes about 2 h for decrosslinking and protein extraction, about 3 h from reductive alkylation to LysC/trypsin digestion, and about 4 h for liquid–liquid extraction using ethyl acetate and C18 clean-up. The time for SWATH measurement is 2 to 3 h per run. These sample processings and measurements are slow compared to the reported rapid protocol based on the urea-PCT-SWATH method^22^. Our protocol can process 16 samples but can only measure 10 samples per day. However, it should be feasible to shorten the run time of SWATH measurement according to the reported rapid protocol^22^.

In conclusion, the present study is the first to establish and validate a quantitative proteomics protocol (highly accurate FFPE-PCT-SWATH) that is able to accurately and comprehensively quantify pathological changes in protein expression levels of not only non-membrane proteins but also membrane proteins, using FFPE samples.

## Supplementary information

Supplementary file1 (PDF 8757 kb)

Supplementary file2 (XLSX 6867 kb)

## Data Availability

The datasets used and/or analyzed during the present study are available from the corresponding author.

## Abbreviations

AAs: Amino acids
BDL: Bile duct ligation
FFPE: Formalin-fixed paraffin-embedded
LC–MS/MS: Liquid chromatography-tandem mass spectrometry
PTS: Phase-transfer surfactant
PCT: Pressure cycling technology
SWATH-MS: Sequential window acquisition of all theoretical fragment ion spectra mass spectrometry
SDC: Sodium deoxycholate
SLS: Sodium lauroylsarcosinate

## References

[1] Steiner C (2014). Applications of mass spectrometry for quantitative protein analysis in formalin-fixed paraffin-embedded tissues. Proteomics.

[2] Shi SR, Taylor CR, Fowler CB, Mason JT (2013). Complete solubilization of formalin-fixed, paraffin-embedded tissue may improve proteomic studies. Proteomics Clin. Appl..

[3] Ostasiewicz P, Zielinska DF, Mann M, Wisniewski JR (2010). Proteome, phosphoproteome, and N-glycoproteome are quantitatively preserved in formalin-fixed paraffin-embedded tissue and analyzable by high-resolution mass spectrometry. J. Proteome Res..

[4] Kennedy JJ (2016). Optimized protocol for quantitative multiple reaction monitoring-based proteomic analysis of formalin-fixed, paraffin embedded tissue. J. Proteome Res..

[5] Fowler CB, Waybright TJ, Veenstra TD, O'Leary TJ, Mason JT (2012). Pressure-assisted protein extraction: a novel method for recovering proteins from archival tissue for proteomic analysis. J. Proteome Res..

[6] Gillet LC (2012). Targeted data extraction of the MS/MS spectra generated by data-independent acquisition: a new concept for consistent and accurate proteome analysis. Mol. Cell Proteomics.

[7] Zhu Y (2019). High-throughput proteomic analysis of FFPE tissue samples facilitates tumor stratification. Mol. Oncol..

[8] Marier JR, Rose D (1964). Determination of cyanate, and a study of its accumulation in aqueous solutions of urea. Anal. Biochem..

[9] Hagel P, Gerding JJ, Fieggen W, Bloemendal H (1971). Cyanate formation in solutions of urea. I. Calculation of cyanate concentrations at different temperature and pH. Biochim. Biophys. Acta.

[10] Masuda T, Tomita M, Ishihama Y (2008). Phase transfer surfactant-aided trypsin digestion for membrane proteome analysis. J. Proteome Res..

[11] Miyauchi E (2018). Identification of blood biomarkers in glioblastoma by SWATH mass spectrometry and quantitative targeted absolute proteomics. PLoS ONE.

[12] Uchida Y (2019). Involvement of claudin-11 in disruption of blood-brain, -spinal cord, and -arachnoid barriers in multiple sclerosis. Mol. Neurobiol..

[13] Wakabayashi M (2014). Phosphoproteome analysis of formalin-fixed and paraffin-embedded tissue sections mounted on microscope slides. J. Proteome Res..

[14] Uchida, Y. *et al.* Abundant expression of OCT2, MATE1, OAT1, OAT3, PEPT2, BCRP, MDR1 and xCT transporters in blood-arachnoid barrier of pig, and polarized localizations at CSF- and blood-facing plasma membranes. *Drug Metab Dispos*, (2020). 10.1124/dmd.119.089516 31771948

[15] Uchida Y, Ohtsuki S, Kamiie J, Terasaki T (2011). Blood-brain barrier (BBB) pharmacoproteomics: reconstruction of in vivo brain distribution of 11 P-glycoprotein substrates based on the BBB transporter protein concentration, in vitro intrinsic transport activity, and unbound fraction in plasma and brain in mice. J. Pharmacol. Exp. Ther.

[16] Long Y (2015). Metabolomics changes in a rat model of obstructive jaundice: mapping to metabolism of amino acids, carbohydrates and lipids as well as oxidative stress. J. Clin. Biochem. Nutr..

[17] Krahenbuhl S, Talos C, Reichen J (1994). Mechanisms of impaired hepatic fatty acid metabolism in rats with long-term bile duct ligation. Hepatology.

[18] Liu Y, Huttenhain R, Collins B, Aebersold R (2013). Mass spectrometric protein maps for biomarker discovery and clinical research. Expert Rev. Mol. Diagn..

[19] Nirmalan NJ (2011). Initial development and validation of a novel extraction method for quantitative mining of the formalin-fixed, paraffin-embedded tissue proteome for biomarker investigations. J. Proteome Res..

[20] Jiang X (2007). Development of efficient protein extraction methods for shotgun proteome analysis of formalin-fixed tissues. J. Proteome Res..

[21] Hamid Z, Summa M, Armirotti A (2018). A swath label-free proteomics insight into the faah(-/-) mouse liver. Sci. Rep..

[22] Gao H (2020). Accelerated lysis and proteolytic digestion of biopsy-level fresh-frozen and FFPE tissue samples using pressure cycling technology. J. Proteome Res..

