# Supplementary material for: Establishment and validation of highly accurate formalin-fixed paraffin-embedded quantitative proteomics by heat-compatible pressure cycling technology using phase-transfer surfactant and SWATH-MS
Source: Sci Rep. 2020 Jul 9;10:11271. doi: 10.1038/s41598-020-68245-2 (PMC7347883; doi:10.1038/s41598-020-68245-2)
Supplement: Supplementary file 1 — Supplementary file1 (PDF 8757 kb) [file 41598_2020_68245_MOESM1_ESM.pdf]

Supplementary materials

Establishment and validation of highly accurate formalin-fixed paraffin-embedded quantitative proteomics by heat-compatible pressure cycling technology using phase-transfer surfactant and SWATH-MS

Yasuo Uchida*, Hayate Sasaki*, and Tetsuya Terasaki

Graduate School of Pharmaceutical Sciences, Tohoku University, Japan

*These two authors equally contributed to this work.

Corresponding author: Yasuo Uchida, Ph.D.
Division of Membrane Transport and Drug Targeting, Graduate School of Pharmaceutical Sciences, Tohoku University, 6-3 Aoba, Aramaki, Aoba-ku, Sendai, 980-8578, Japan.
Voice: +81-22-795-6832; FAX: +81-22-795-6886;
E-mail: yasuo.uchida.c8@tohoku.ac.jp

1. Supplementary figures 1 to 12
2. Supplementary tables 1 to 14 (uploaded as an Excel file)
3. Supplementary methods

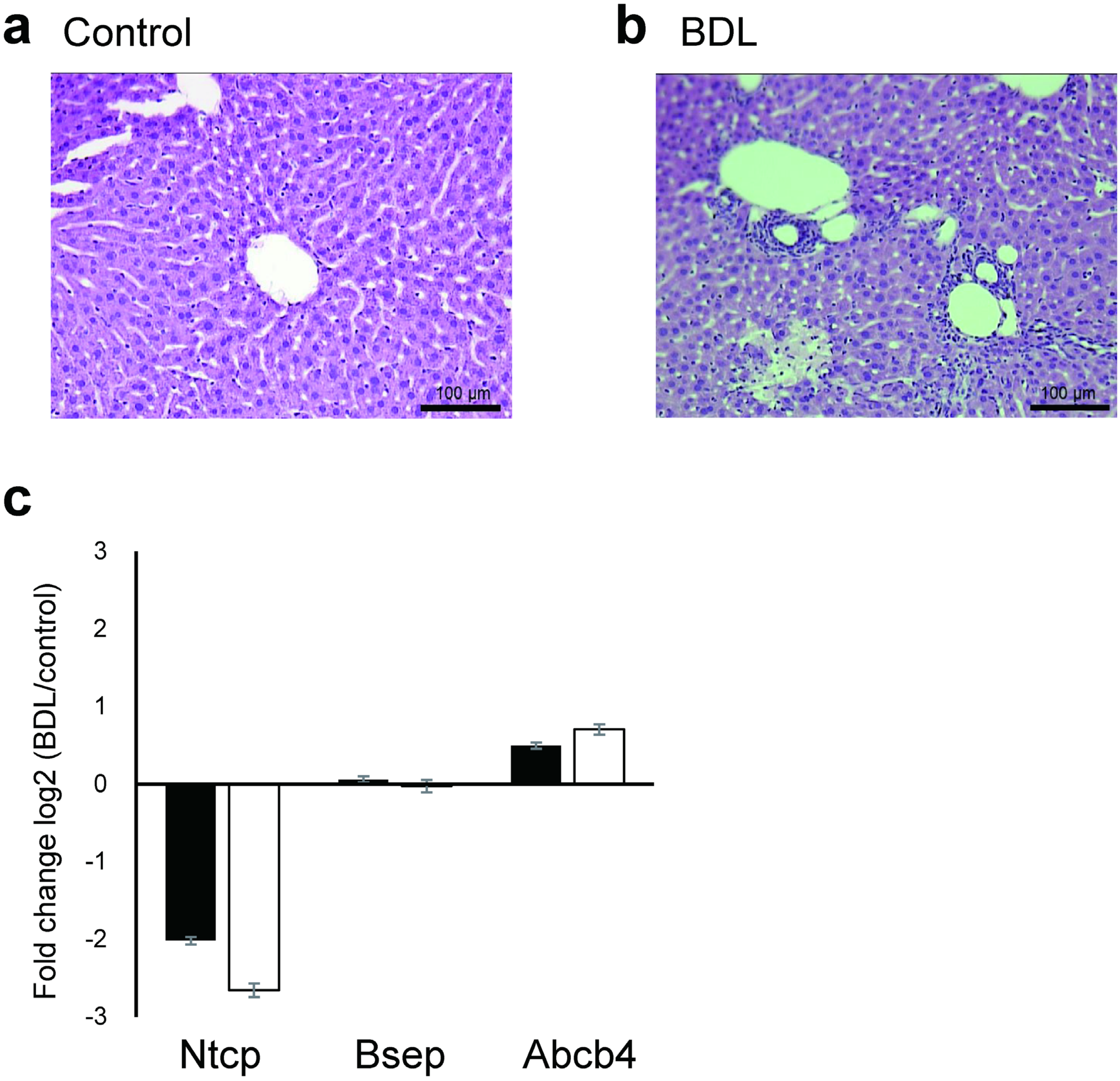

Supplementary figure 1 Comparison of the BDL model mouse generated in the present study with that in the previous study

[a, and b] 5 µm FFPE sections of control (a) and BDL (b) mouse liver were stained with hematoxylin and eosin for pathology review. The scale bar in each figure panel represents 100 µm. **[c]** Changes in the expression levels of three transporters (Ntcp, Bsep and Abcb4) induced by BDL in the present study were compared with those in the previous report.[1] The whole tissue lysate of mouse fresh liver was processed by PCT and the peptide samples were subjected to SWATH analysis. Closed and open columns represent the fold changes in the present study and the previous study, respectively. Each column represents the mean ± SEM (n=4 for control; n=3-4 for BDL). The SEM value was calculated according to the law of propagation of error, as previously reported.[2]

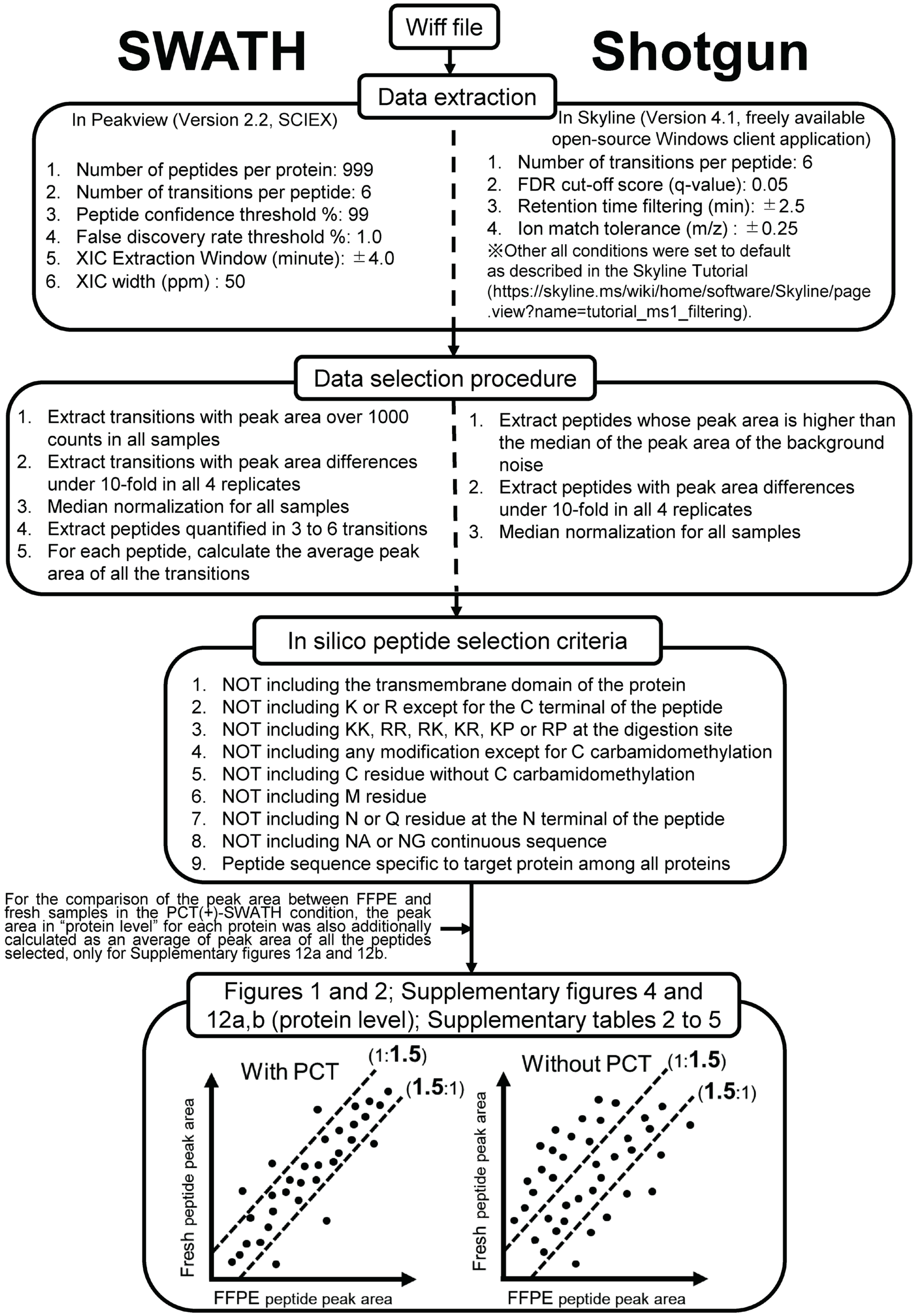

Supplementary figure 2 Data analysis workflow for SWATH and shotgun measurements

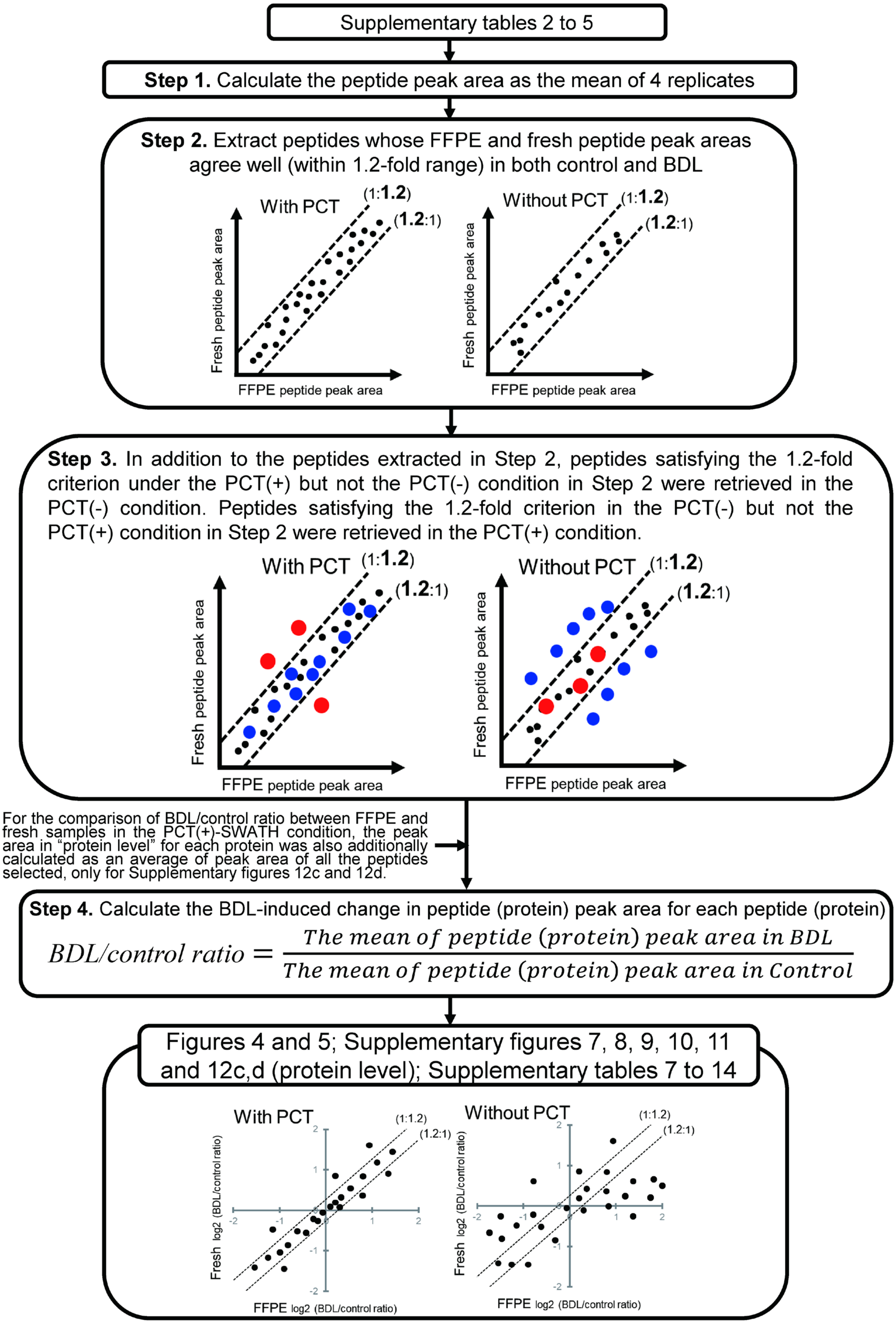

Supplementary figure 3　Data analysis workflow for the evaluation of BDL-induced changes in protein expression level

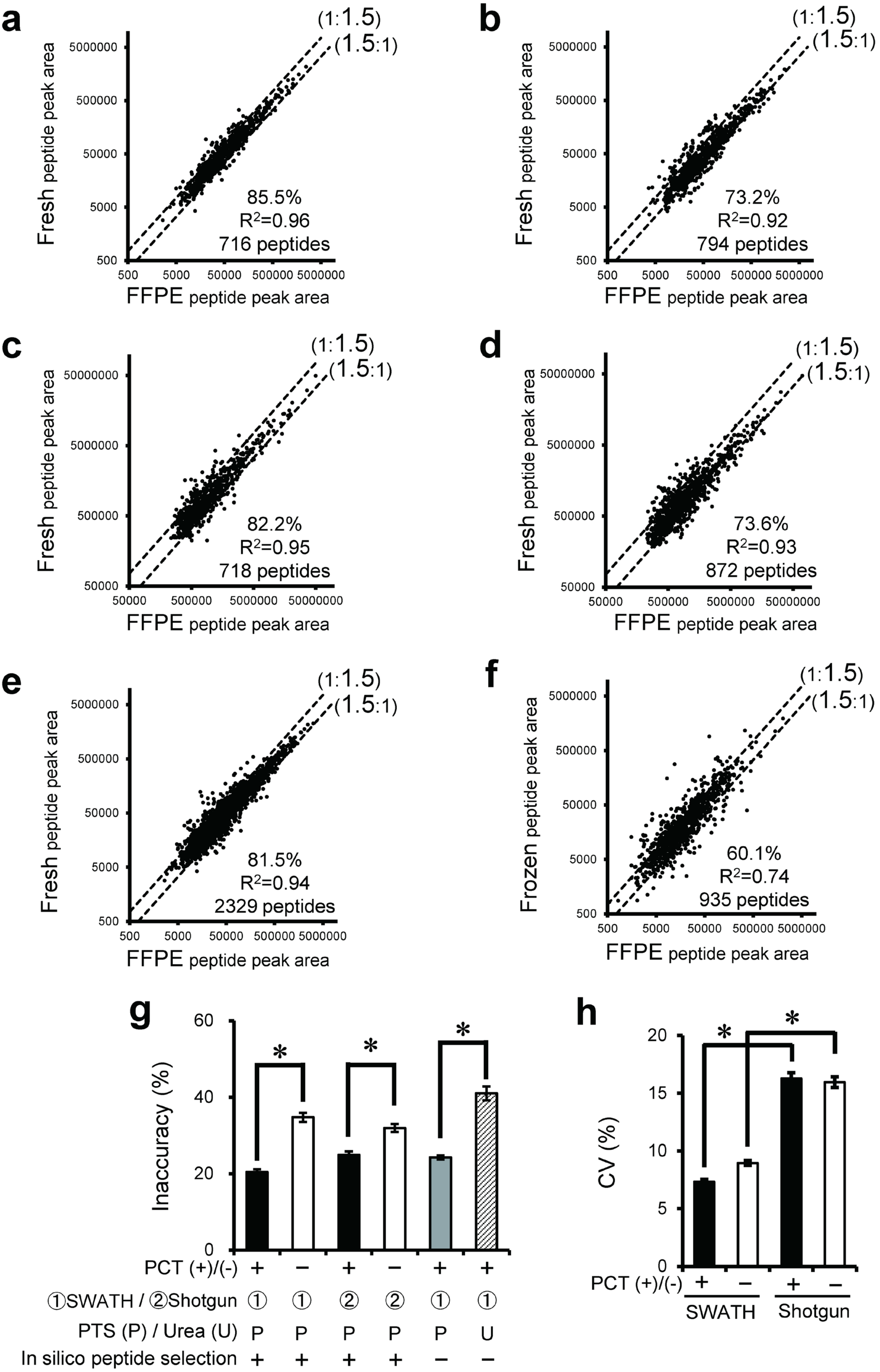

a
(1:1.5)
(1.5:1)
Fresh peptide peak area
FFPE peptide peak area
85.5%
R2=0.96
716 peptides
b
(1:1.5)
(1.5:1)
Fresh peptide peak area
FFPE peptide peak area
73.2%
R2=0.92
794 peptides
c
(1:1.5)
(1.5:1)
Fresh peptide peak area
FFPE peptide peak area
82.2%
R2=0.95
718 peptides
d
(1:1.5)
(1.5:1)
Fresh peptide peak area
FFPE peptide peak area
73.6%
R2=0.93
872 peptides
e
(1:1.5)
(1.5:1)
Fresh peptide peak area
FFPE peptide peak area
81.5%
R2=0.94
2329 peptides
f
(1:1.5)
(1.5:1)
Frozen peptide peak area
FFPE peptide peak area
60.1%
R2=0.74
935 peptides
g
Inaccuracy (%)
PCT (+)/(-) + − + − + +
①SWATH / ②Shotgun ① ① ② ② ① ①
PTS (P) / Urea (U) P P P P P U
In silico peptide selection + + + + − −
h
CV (%)
PCT (+)/(-) + − + −
SWATH
Shotgun

Supplementary figure 4 Effect of PCT treatment, SWATH analysis, heat-compatible PTS buffer and in silico peptide selection criteria on the comprehensive quantification of cytosolic proteins in FFPE sections of mouse liver

[a, b, c, d and e] Cytosolic proteins were selected according to the subcellular location information in the Uniprot mouse proteome database. Peptide peak areas were compared between FFPE and fresh mouse liver samples. Peptides were prepared with PCT treatment using PTS buffer (95 °C heating for lysis) and measured in the SWATH mode [a and e, PCT(+)-SWATH], or prepared without PCT treatment (only incubation in PTS buffer at 95 °C for lysis) and measured in the SWATH mode [b, PCT(-)-SWATH], or prepared with PCT treatment using PTS buffer (95 °C heating for lysis) and measured in the shotgun mode [c, PCT(+)-Shotgun], or prepared without PCT treatment (only incubation in PTS buffer at 95 °C for lysis) and measured in the shotgun mode [d, PCT(-)-Shotgun]. Data analysis for a, b, c and d was carried out as described in Supplementary figure 2 with in silico peptide selection criteria. The data were taken from Supplementary tables 2, 3, 4 and 5. Data analysis for e was carried out as shown in Supplementary figure 2 but without application of the in silico peptide selection criteria. Each point represents the mean (n=4). The broken lines represent 1.5-fold difference. The % in each scatter plot is the proportion of peptides whose peak areas from FFPE are consistent with those from fresh samples within a 1.5-fold range. **[f]** This graph was generated using the data of the reported PCT-SWATH study[3]. Cytosolic proteins were selected according to the subcellular location information in the Uniprot human proteome database. Peptide peak areas were compared between FFPE and frozen human benign prostatic tissues obtained from the same resected tissue of patient 15, who showed the best agreement (as an inaccuracy value) between FFPE and frozen peptide peak areas among the 24 patients. The peptide samples of FFPE and frozen prostatic tissues were prepared with PCT treatment using urea buffer without heating, and then measured in the SWATH mode. The in silico peptide selection criteria were not applied. The broken lines represent 1.5-fold differences. The % in each scatter plot is the proportion of peptides whose peak areas from FFPE samples lie within a 1.5-fold range of those from frozen samples. **[g, and h]** Inaccuracy (g) and CV values (h) corresponding to panels a, b, c, and d were calculated as described in Materials & Methods, and the inaccuracy (g) was also calculated from panels e (gray column) and f (hatched column). Each column represents the mean ± SEM (n = 716-2329 peptides; number commonly detected in FFPE and fresh samples under each experimental condition). *$p < 0.001$, significant difference between two groups (Bonferroni-corrected Student's *t*-test).

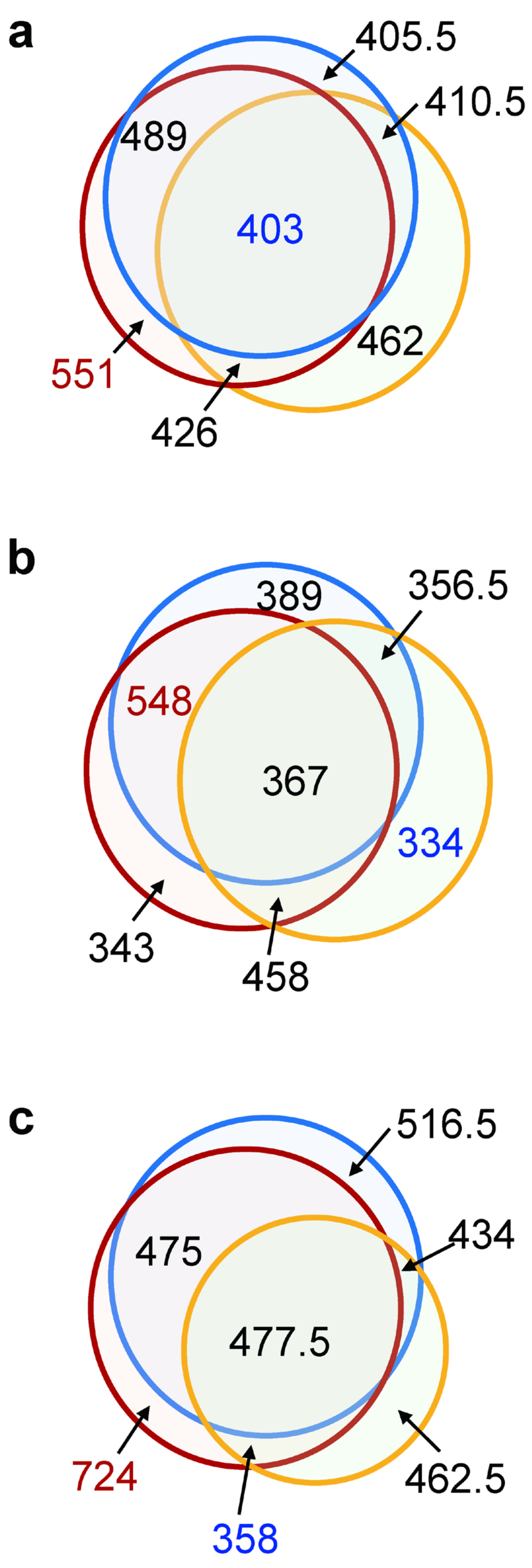

Supplementary figure 5 The median numbers of amino acid residues (AAs) of proteins in each region of the Venn diagrams in Figures 3a, 3b and 3c

a (total), b (cytosolic) and c (membrane) correspond to Figures 3a, 3b and 3c, respectively. The median numbers of AAs are shown. Red and blue numbers represent the highest and lowest medians in each case. The number of AAs was obtained from the Uniprot database.

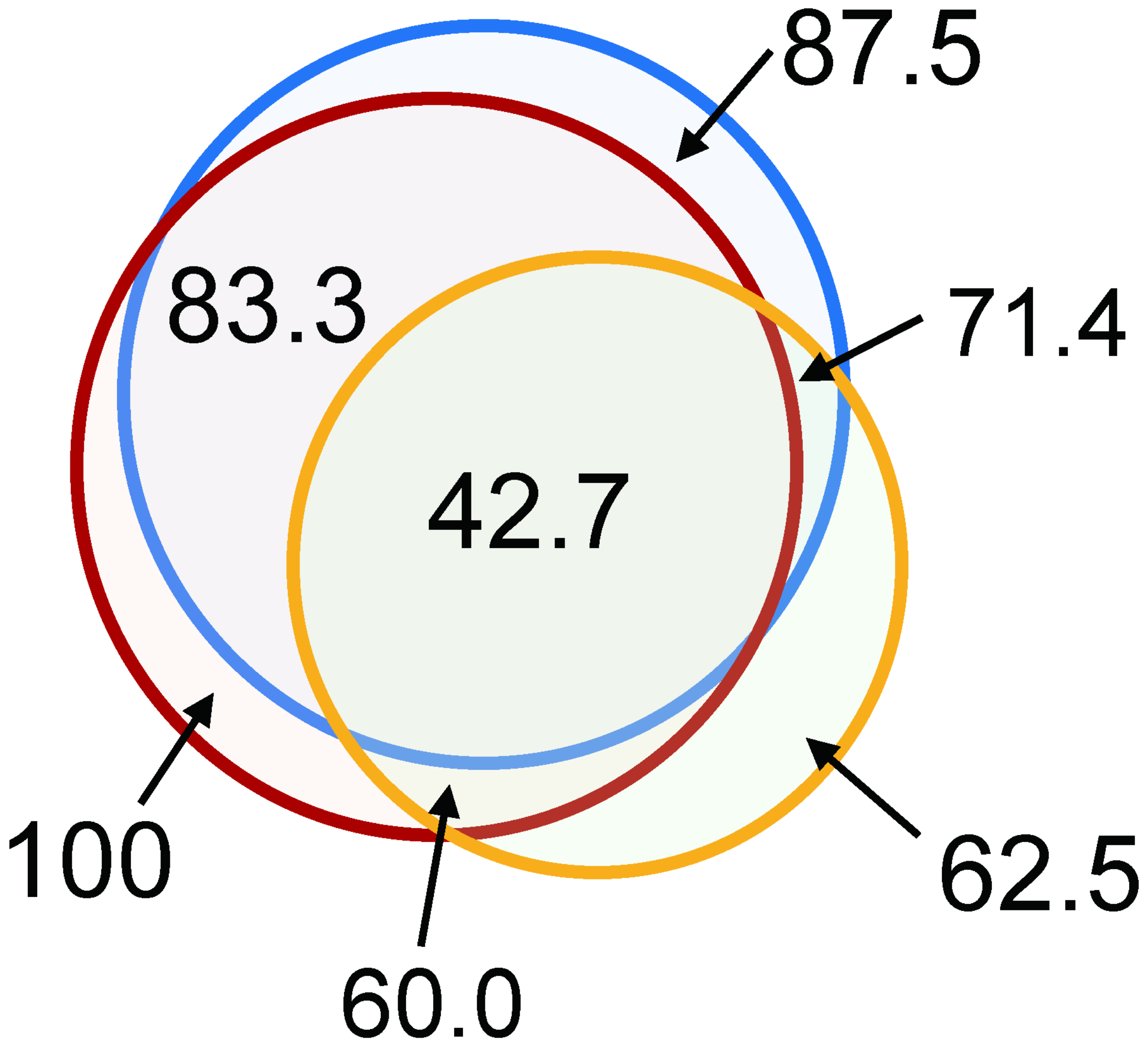

Supplementary figure 6 The percentage of transmembrane proteins among membrane proteins in each region of the Venn diagram in Figure 3c

The presence or absence of a transmembrane region in each membrane protein was established from the Uniprot database.

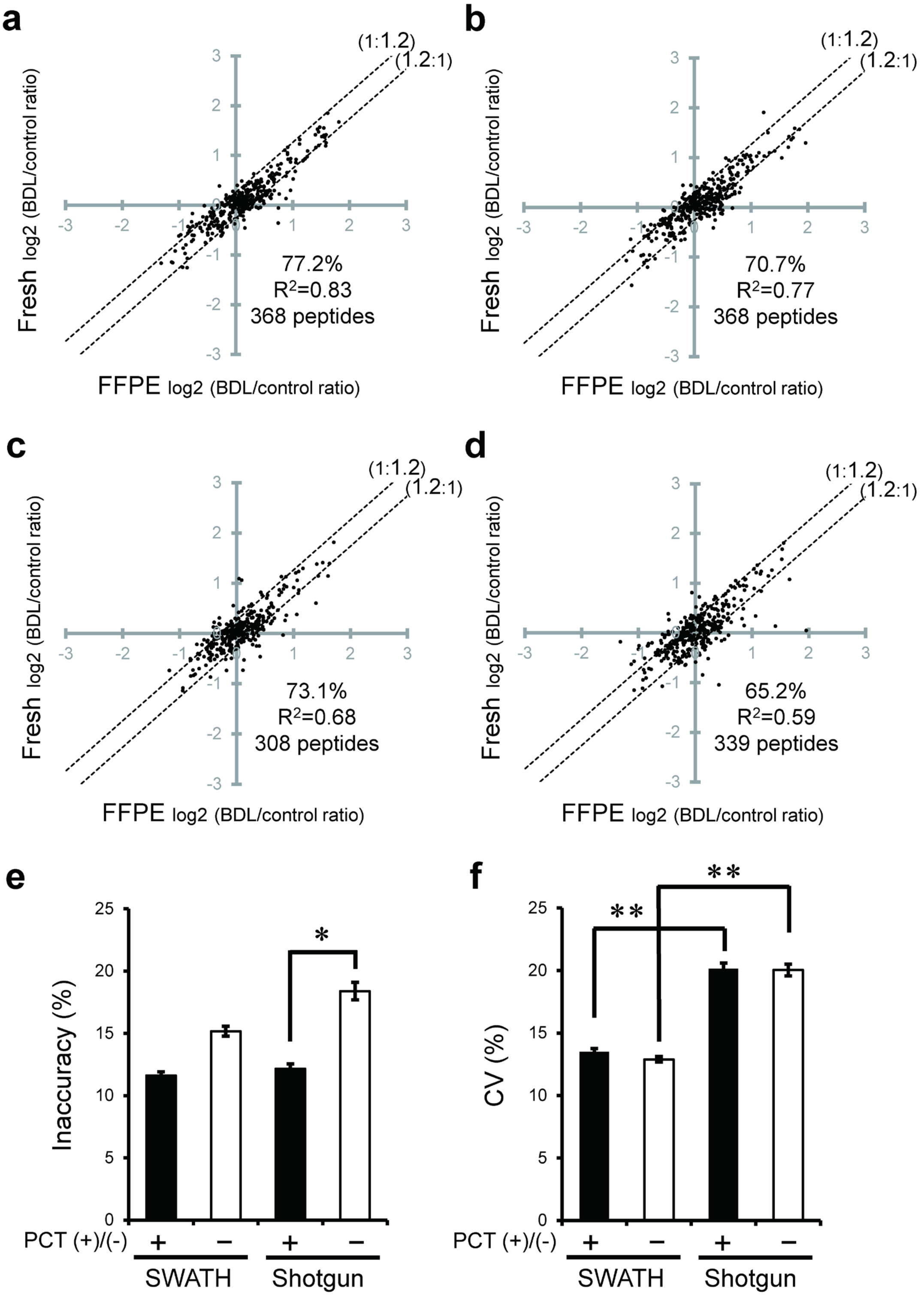

Supplementary figure 7 Effect of PCT treatment and SWATH analysis on comprehensive quantification of BDL-induced changes in expression levels of cytosolic proteins in FFPE sections

[a, b, c, and d] Cytosolic proteins were selected according to the subcellular location information in the Uniprot mouse proteome database. The BDL-induced changes in expression level (BDL/control ratio) were compared between FFPE and fresh samples of mouse liver at the peptide level. Peptide samples from FFPE and fresh livers were prepared with PCT treatment and measured in the SWATH mode (a, PCT(+)-SWATH), without PCT treatment and measured in the SWATH mode (b, PCT(-)-SWATH), with PCT treatment and measured in the shotgun mode (c, PCT(+)-Shotgun), or without PCT treatment and measured in the shotgun mode (d, PCT(-)-Shotgun). Data analysis was carried out as described in Supplementary figure 3. The data were taken from Supplementary tables 7, 8, 9 and 10. Each point represents the mean (n=4). The broken lines indicate 1.2-fold differences. The % in each scatter plot is the proportion of peptides whose peak areas from FFPE are consistent with those from fresh samples within a 1.2-fold range. **[e, and f]** Inaccuracy (e) and CV value (f) corresponding to panels a, b, c, and d were calculated as described in Materials & Methods. Each column represents the mean ± SEM (n=308-368 peptides; number commonly detected in FFPE and fresh samples under each experimental condition). **$p < 0.001$, *$p < 0.01$, significant difference between two groups (Bonferroni-corrected Student's t-test).

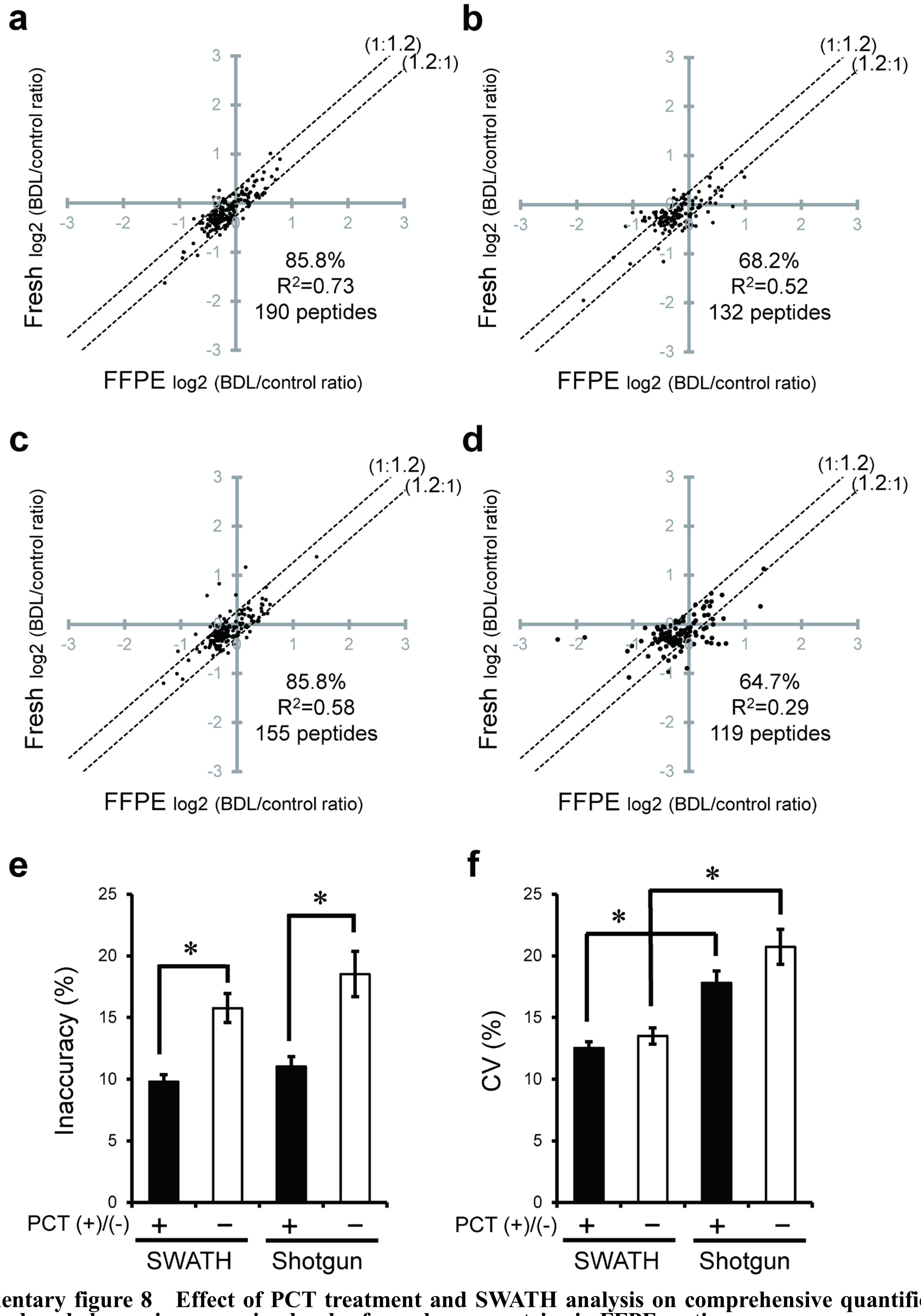

Supplementary figure 8 Effect of PCT treatment and SWATH analysis on comprehensive quantification of BDL-induced change in expression levels of membrane proteins in FFPE sections

[a, b, c, and d] Membrane proteins were selected according to the subcellular location information in the Uniprot mouse proteome database. The BDL-induced changes in expression level (BDL/control ratio) were compared between FFPE and fresh samples of mouse liver at the peptide level. Peptide samples of FFPE and fresh livers were prepared with PCT treatment and measured in the SWATH mode (a, PCT(+)-SWATH), without PCT treatment and measured in the SWATH mode (b, PCT(-)-SWATH), with PCT treatment and measured in the shotgun mode (c, PCT(+)-Shotgun), or without PCT treatment and measured in the shotgun mode (d, PCT(-)-Shotgun). Data analysis was carried out as described in Supplementary figure 3. The data were taken from Supplementary tables 7, 8, 9 and 10. Each point represents the mean (n=4). The broken lines indicate 1.2-fold differences. The % in each scatter plot is the proportion of peptides whose peak areas from FFPE are consistent with those from fresh samples within a 1.2-fold range. **[e, and f]** Inaccuracy (e) and CV value (f) corresponding to panels a, b, c, and d were calculated as described in Materials & Methods. Each column represents the mean ± SEM (n=119-190 peptides; number commonly detected in FFPE and fresh samples under each experimental condition). *$p < 0.001$, significant difference between two groups (Bonferroni-corrected Student's t-test).

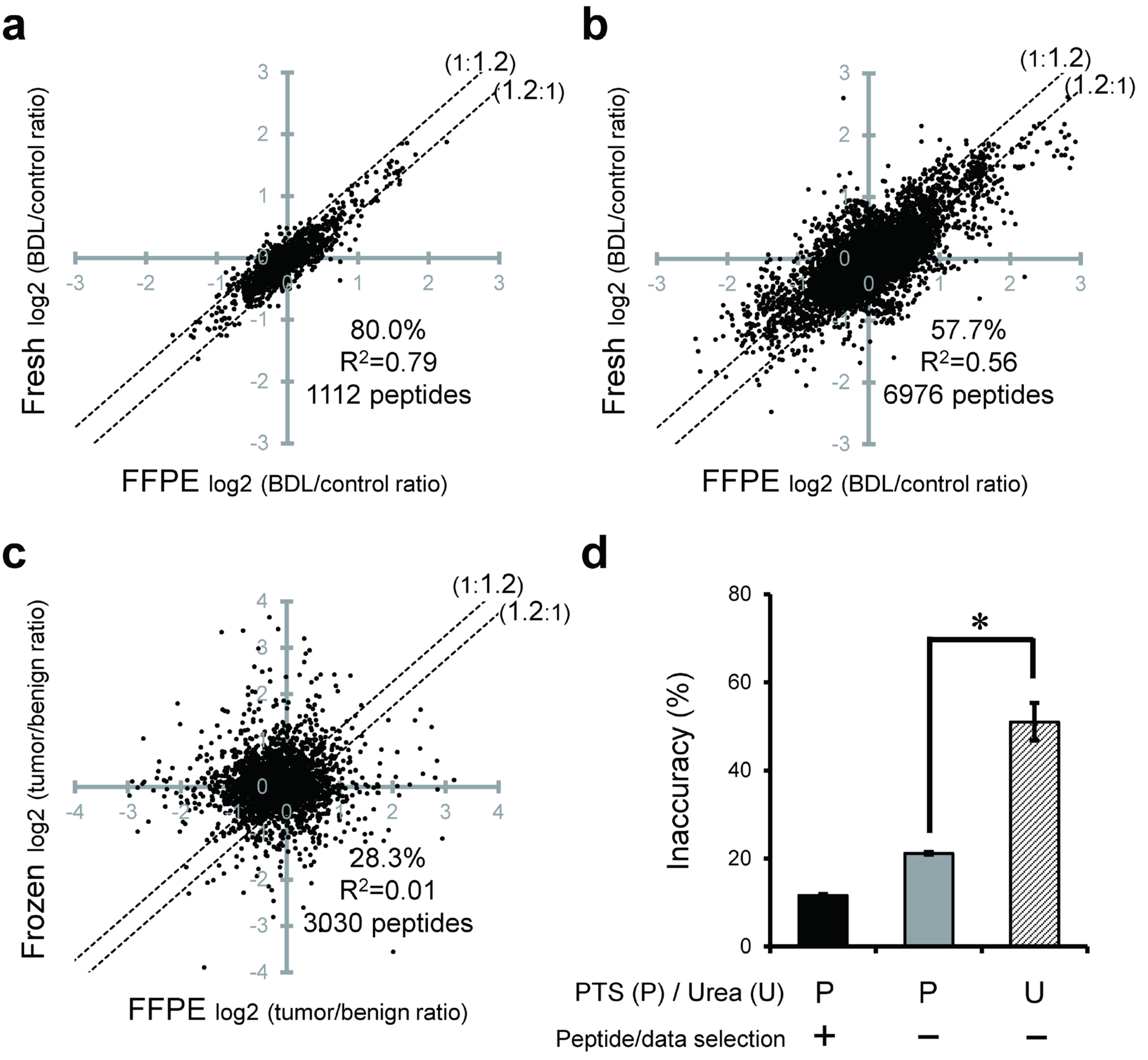

Supplementary figure 9 Effect of heat-compatible PTS buffer and peptide/data selection criteria on the comprehensive quantification of pathological changes in protein expression level in FFPE tissues
[a, and b] The BDL-induced changes in expression level (BDL/control ratio) were compared between FFPE and fresh samples of mouse liver at the peptide level. Peptide samples of FFPE and fresh livers were prepared with PCT treatment using PTS buffer (95 °C heating for lysis) and measured in the SWATH mode [PCT(+)-SWATH]. Data analysis was conducted with in silico peptide selection criteria and the data selection illustrated in Supplementary figure 3 (a) or without the peptide and data selections (b). Each point represents the mean (n=4). The broken lines indicate 1.2-fold differences. The % in each scatter plot is the proportion of peptides whose peak areas from FFPE are consistent with those from fresh samples within a 1.2-fold range. **[c]** This graph was generated using the data from the reported PCT-SWATH study[3] for FFPE and frozen tumorous and benign prostatic tissues. Tumor/benign ratios were compared between FFPE and frozen tissues obtained from the same resected tissues of patient 15, who showed the best agreement (as an inaccuracy value) between FFPE and frozen tumor/benign ratios among the 24 patients. The peptide samples of FFPE and frozen prostatic tissues were prepared with PCT treatment using urea buffer without heating, and then measured in the SWATH mode. The in silico peptide selection criteria and the data selection illustrated in Supplementary figure 3 were not applied. The broken lines indicate 1.2-fold differences. The % in each scatter plot is the proportion of peptides whose peak areas from FFPE are consistent with those from frozen samples within a 1.2-fold range. **[d]** Inaccuracy values corresponding to panels a, b and c were calculated as described in Materials & Methods. Each column represents the mean ± SEM (n=1112-6976 peptides; number commonly detected in FFPE and fresh (frozen) samples under each experimental condition). *$p < 0.001$, significant difference between two groups (Bonferroni-corrected Student's t-test).

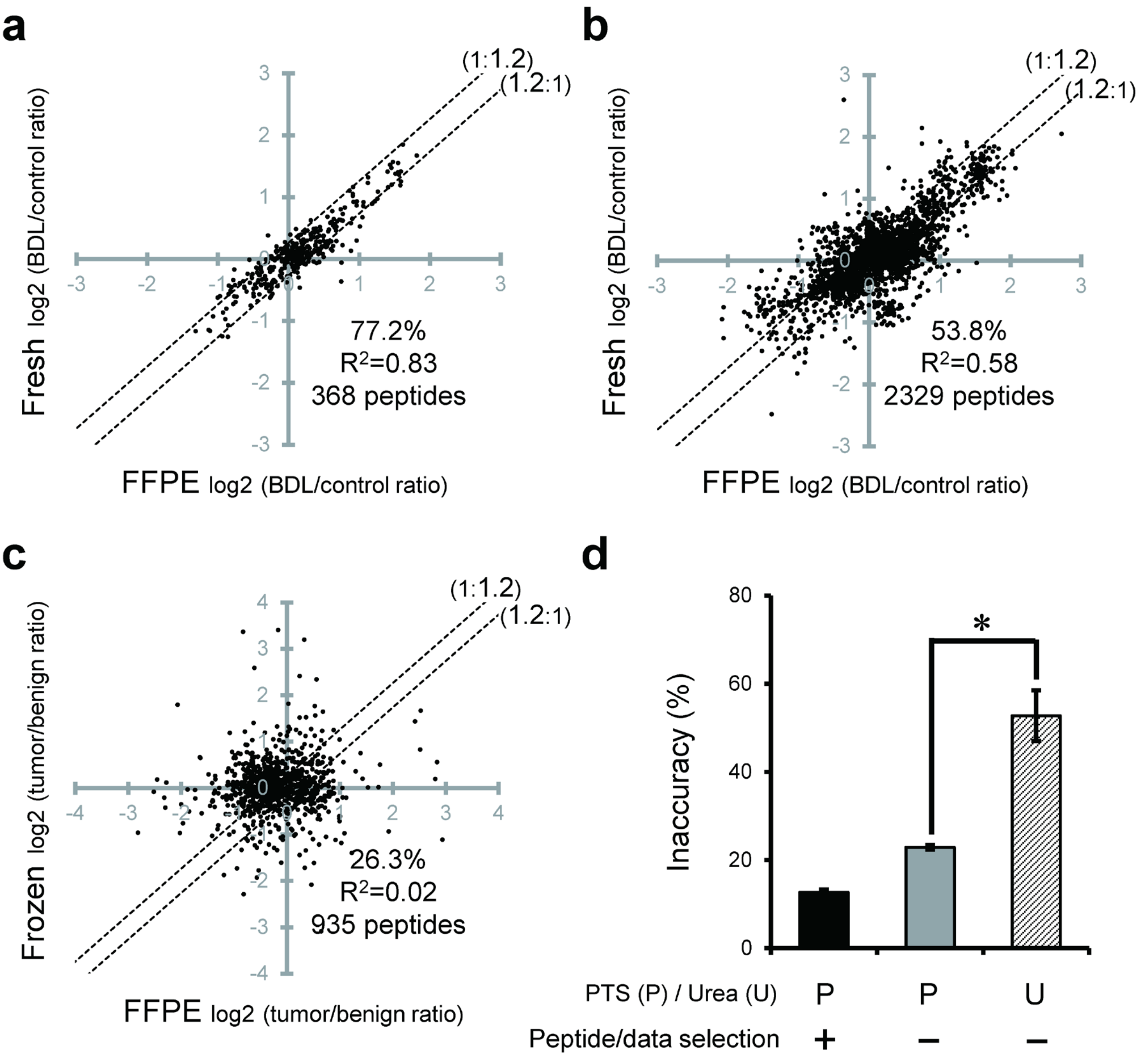

Supplementary figure 10 Effect of heat-compatible PTS buffer and peptide/data selection criteria on the comprehensive quantification of pathological changes in expression levels of cytosolic proteins in FFPE tissues

[a, and b] Cytosolic proteins were selected according to the subcellular location information in the Uniprot mouse proteome database. The BDL-induced changes in expression level (BDL/control ratio) were compared between FFPE and fresh samples of mouse liver at the peptide level. Peptide samples of FFPE and fresh livers were prepared with PCT treatment using PTS buffer (95 °C heating for lysis) and measured in the SWATH mode [PCT(+)-SWATH]. Data analysis was conducted with in silico peptide selection criteria and the data selection illustrated in Supplementary figure 3 (a) or without the peptide and data selections (b). Each point represents the mean (n=4). The broken lines indicate 1.2-fold differences. The % in each scatter plot is the proportion of peptides whose peak areas from FFPE are consistent with those from fresh samples within a 1.2-fold range. **[c]** This graph was generated using the data from the reported PCT-SWATH study[3] for FFPE and frozen tumorous and benign prostatic tissues. Cytosolic proteins were selected according to the subcellular location information in the Uniprot human proteome database. Tumor/benign ratios were compared between FFPE and frozen tissues obtained from the same resected tissues of patient 15, who showed the best agreement (as an inaccuracy value) between FFPE and frozen tumor/benign ratios among the 24 patients. The peptide samples of FFPE and frozen prostatic tissues were prepared with PCT treatment using urea buffer without heating, and then measured in the SWATH mode. The in silico peptide selection criteria and the data selection illustrated in Supplementary figure 3 were not applied. The broken lines indicate 1.2-fold differences. The % in each scatter plot is the proportion of peptides whose peak areas from FFPE are consistent with those from frozen samples within a 1.2-fold range. **[d]** Inaccuracy values corresponding to panels a, b and c were calculated as described in Materials & Methods. Each column represents the mean ± SEM (n=368-2329 peptides; number commonly detected in FFPE and fresh (frozen) samples under each experimental condition). *$p < 0.001$, significant difference between two groups (Bonferroni-corrected Student's t-test).

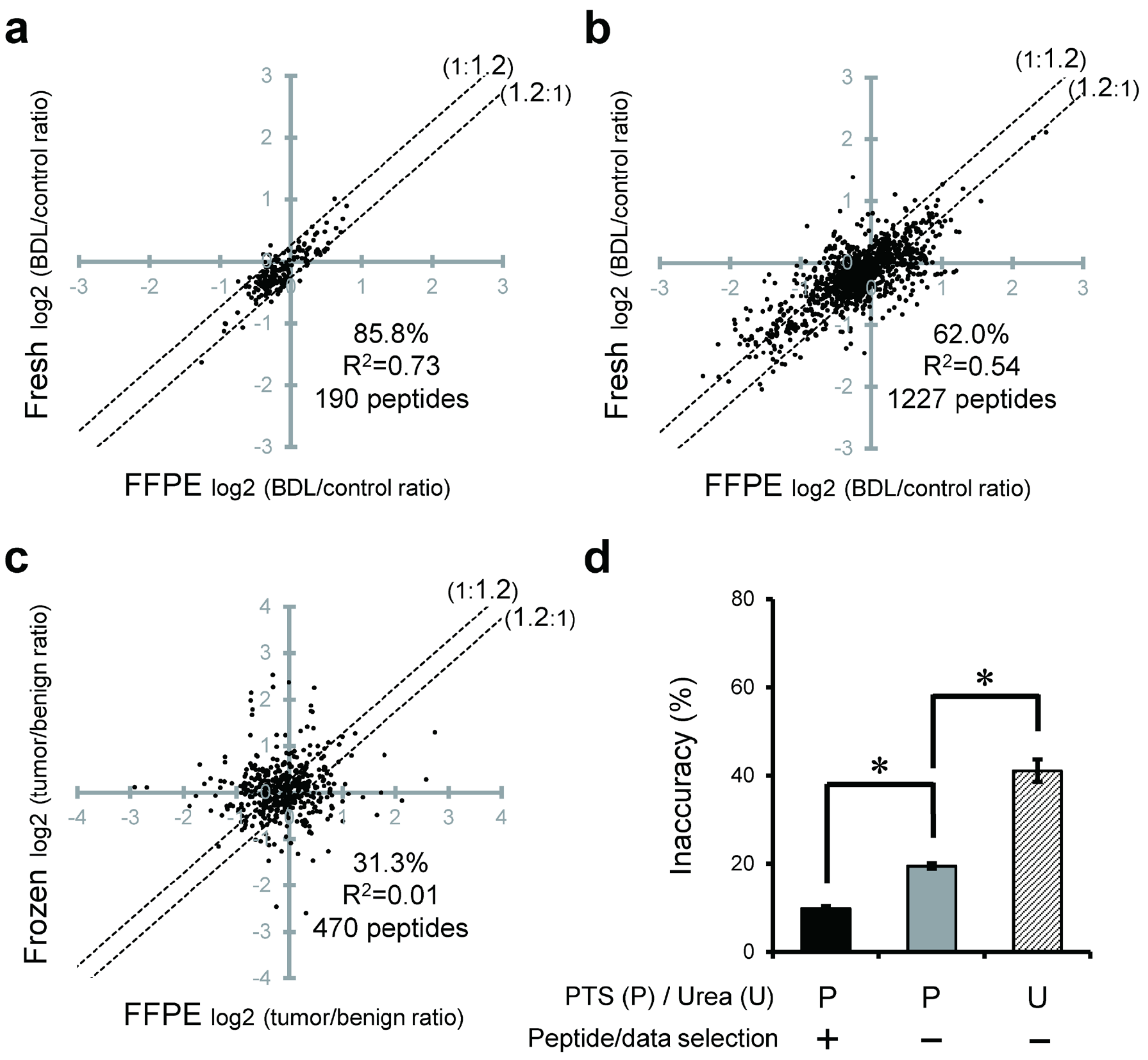

Supplementary figure 11 Effect of heat-compatible PTS buffer and peptide/data selection criteria on the comprehensive quantification of pathological changes in expression levels of membrane proteins in FFPE tissues

[a, and b] Membrane proteins were selected according to the subcellular location information in the Uniprot mouse proteome database. The BDL-induced changes in expression level (BDL/control ratio) were compared between FFPE and fresh samples of mouse liver at the peptide level. Peptide samples of FFPE and fresh livers were prepared with PCT treatment using PTS buffer (95 °C heating for lysis) and measured in the SWATH mode [PCT(+)-SWATH]. Data analysis was conducted with in silico peptide selection criteria and the data selection illustrated in Supplementary figure 3 (a) or without the peptide and data selections (b). Each point represents the mean (n=4). The broken lines indicate 1.2-fold differences. The % in each scatter plot is the proportion of peptides whose peak areas from FFPE are consistent with those from fresh samples within a 1.2-fold range. **[c]** This graph was generated using the data from the reported PCT-SWATH study[3] for FFPE and frozen tumorous and benign prostatic tissues. Membrane proteins were selected according to the subcellular location information in the Uniprot human proteome database. Tumor/benign ratios were compared between FFPE and frozen tissues obtained from the same resected tissues of patient 15, who showed the best agreement (as an inaccuracy value) between FFPE and frozen tumor/benign ratios among the 24 patients. The peptide samples of FFPE and frozen prostatic tissues were prepared with PCT treatment using urea buffer without heating, and then measured in the SWATH mode. The in silico peptide selection criteria and the data selection illustrated in Supplementary figure 3 were not applied. The broken lines indicate 1.2-fold differences. The % in each scatter plot is the proportion of peptides whose peak areas from FFPE are consistent with those from frozen samples within a 1.2-fold range. **[d]** Inaccuracy values corresponding to panels a, b and c were calculated as described in Materials & Methods. Each column represents the mean ± SEM (n=190-1227 peptides; number commonly detected in FFPE and fresh (frozen) samples under each experimental condition). *$p < 0.001$, significant difference between two groups (Bonferroni-corrected Student's t-test).

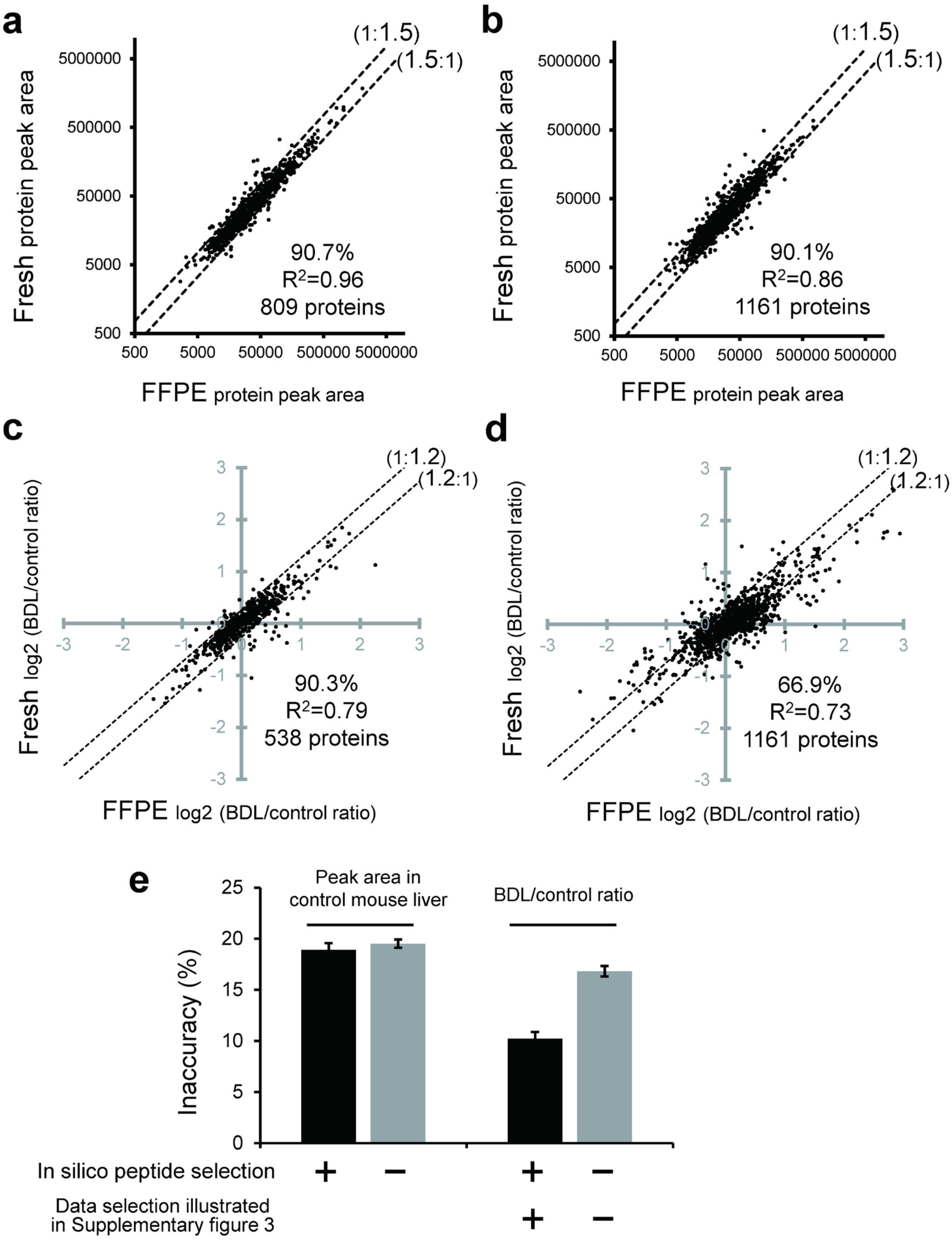

Supplementary figure 12 Comparison of peak area and BDL/control ratio between FFPE and fresh samples using protein level data

[a, and b] Protein peak areas were compared between FFPE and fresh mouse livers. Peptide samples of FFPE and fresh livers were prepared with PCT treatment using PTS buffer (95 °C heating for lysis) and measured in the SWATH mode [PCT(+)-SWATH]. Data analysis was conducted as described in Supplementary figure 2 with the calculation of protein peak areas as the average values of the peptide peak areas for each protein, with (a) or without (b) in silico peptide selection criteria. Each point represents the mean (n = 4). The broken lines represent 1.5-fold differences. The % in each scatter plot is the proportion of proteins whose peak areas from FFPE samples lie within a 1.5-fold range of those from fresh samples. **[c, and d]** The BDL-induced changes in expression level (BDL/control ratio) were compared between FFPE and fresh samples of mouse liver at the protein level. Peptide samples of FFPE and fresh livers were prepared with PCT treatment using PTS buffer (95 °C heating for lysis) and measured in the SWATH mode [PCT(+)-SWATH]. Data analysis was conducted as described in Supplementary figure 3 with the calculation of protein peak areas as average values of peptide peak areas for each protein, with in silico peptide selection criteria and the data selection illustrated in Supplementary figure 3 (c) or without the peptide and data selections (d). Each point represents the mean (n=4). The broken lines indicate 1.2-fold differences. The % in each scatter plot is the proportion of proteins whose peak areas from FFPE are consistent with those from fresh samples within a 1.2-fold range. **[e]** Inaccuracy values corresponding to panels a, b, c and d were calculated as described in Materials & Methods. Each column represents the mean ± SEM (n=538-1161 proteins; number commonly detected in FFPE and fresh samples under each experimental condition).

Supplementary methods

Normal and bile duct ligation (BDL) mice

Male C57BL/6 mice (7-8 weeks of age) were purchased from Japan SLC (Hamamatsu, Japan). All experimental protocols were approved by the Institutional Animal Care and Use Committee in Tohoku University, and were performed in accordance with the guidelines of Tohoku University. The bile duct ligation (BDL) mouse model was prepared as described previously.[1] Briefly, under anesthesia induced with isoflurane, the abdominal cavity was opened and the common bile duct was dissected, and doubly ligated with commercial surgical sutures, leaving the gall bladder intact. The abdominal muscle and wound were sutured with commercial surgical sutures. The liver was isolated at 7 days after BDL. We confirmed that the BDL model mice had been properly prepared by staining the liver with hematoxylin and eosin (H&E); pathological review revealed liver damage, as expected (Supplementary figure 1). In addition, the changes in expression levels of ntcp, bsep (bile acids transporters) and abcb4 (phospholipid transporter), measured in the SWATH mode, were consistent with those reported previously[1] (Supplementary figure 1).

Evaluation of PCT-assisted protein extraction from FFPE sections

The effect of PCT on protein extraction from FFPE sections was evaluated by comparison with the extraction of proteins from frozen sections into PTS buffer. The FFPE tissue suspension prepared as described in Materials & Methods was used. For the frozen samples, mouse liver in OCT compound was sectioned in a cryostat at −20 °C, and the sections were suspended in PTS buffer by sonication, so that the tissue concentration ([number of sections] × [thickness per section] × [area per section] / [volume of PTS buffer]) was the same as that of the FFPE suspension. Aliquots of 120 µL of FFPE and frozen suspensions were placed in PCT Micro Tubes (Pressure BioSciences, South Easton, MA) with PCT Micro Caps (100 µL size) (Pressure BioSciences, South Easton, MA), and processed with or without PCT. The protein concentrations in the solution were determined by means of BCA assay. The protocol is described below.

Preparation of FFPE tissue suspension was carried out as described in Materials & Methods. To verify the efficiency of the extraction, we also prepared matched frozen sections from mouse liver as follows. Frozen mouse liver samples were anchored in OCT compound, and twenty 10 µm sections were cut on a cryostat at −20 °C. All the sections were added to 4.8 mL of PTS buffer in a 15 mL low protein adsorption centrifuge tube, and the sample was suspended by sonication. The ratio of tissue volume to buffer volume is theoretically the same in the FFPE and frozen tissue suspensions. Aliquots of 120 µL of FFPE and frozen homogenates were placed in PCT Micro Tubes (Pressure BioSciences, South Easton, MA) with PCT Micro Caps (100 µL size) (Pressure BioSciences, South Easton, MA). All samples were incubated at 95°C for 60 min in block incubator (Eppendorf, Hamburg, Germany) with mixing at 1000 rpm. Thereafter, both FFPE and frozen samples were processed in two ways, with or without PCT treatment. For PCT treatment,

samples were incubated in a Barocycler (NEP 2320 Enhanced; Pressure BioSciences, South Easton, MA) in two steps as follows: firstly 60 cycles of 95 seconds at 45000 psi and 5 seconds at atmospheric pressure at 95 °C, and secondly 50 cycles of 20 seconds at 45000 psi and 15 seconds at atmospheric pressure at 95 °C. Samples without PCT treatment were simply incubated in the block incubator (Eppendorf, Hamburg, Germany; without mixing) at 95 °C for the same time as the PCT treatment. Finally, all samples were cooled to room temperature, and centrifuged at 15000 rpm for 3 min. The protein concentration in the supernatant was determined by means of BCA assay.

Generation of the spectral library for SWATH analysis

The tryptic digests of FFPE tissue suspension of mouse liver were fractionated by isoelectric focusing and measured in the shotgun mode as described previously.[4] Shotgun data were analyzed using the Paragon algorithm of ProteinPilot Version 4.5 (SCIEX), and the UniProt Mouse proteome database (release2018_03, entries) was searched. The six user-defined options were: (i) cysteine alkylation, iodoacetamide; (ii) digestion, trypsin digestion; (iii) special factors, none; (iv) species, *Mus musculus*; (v) identification focus, biological modification; and (vi) search effort, thorough identification search. The identification confidence is >99%. The FDR values were all lower than 1%. Finally, excluding overlapping peptides or proteins, 30179 peptides and 3140 proteins were included in the spectral library, which was uploaded in the PeptideAtlas webpage with identifier PASS01457.

References

1 Sasaki, K. *et al.* ATP-Binding Cassette Transporter A Subfamily 8 Is a Sinusoidal Efflux Transporter for Cholesterol and Taurocholate in Mouse and Human Liver. *Mol Pharm* **15**, 343-355 (2018).

2 Uchida, Y., Ohtsuki, S., Kamiie, J. & Terasaki, T. Blood-brain barrier (BBB) pharmacoproteomics: reconstruction of in vivo brain distribution of 11 P-glycoprotein substrates based on the BBB transporter protein concentration, in vitro intrinsic transport activity, and unbound fraction in plasma and brain in mice. *J Pharmacol Exp Ther* **339**, 579-588 (2011).

3 Zhu, Y. *et al.* High-throughput proteomic analysis of FFPE tissue samples facilitates tumor stratification. *Mol Oncol* **13**, 2305-2328 (2019).

4 Uchida, Y. *et al.* Involvement of Claudin-11 in Disruption of Blood-Brain, -Spinal Cord, and -Arachnoid Barriers in Multiple Sclerosis. *Mol Neurobiol* **56**, 2039-2056 (2019).
